# The central role of microglia in major depressive disorder and its potential as a therapeutic target

**DOI:** 10.3389/fnbeh.2025.1598178

**Published:** 2025-08-11

**Authors:** Xue Xia, Kaiqing Li, Wei Zou, Long Wang

**Affiliations:** ^1^Department of Graduate School of Heilongjiang University of Chinese Medicine, Harbin, China; ^2^Department of Acupuncture and Moxibustion, The First Affiliated Hospital of Heilongjiang University of Traditional Chinese Medicine, Harbin, China

**Keywords:** microglia, major depressive disorder, pathogenesis, immunity, neuroinflammation, therapeutic targets

## Abstract

Major depressive disorder (MDD) is a complex neuropsychiatric condition whose multifactorial etiology remains incompletely explained by neuron-centric and neurotransmitter hypotheses alone. This review addresses that gap by positioning microglia—the CNS’s resident immune cells—as central drivers of MDD pathogenesis. We organize current evidence around five interrelated themes: hypothalamic–pituitary–adrenal (HPA) axis dysfunction, monoaminergic and kynurenine pathway imbalances, neuroinflammatory overactivation, synaptic and white-matter integrity disruption, and gut–brain axis perturbations. In MDD, microglia shift from a surveillant resting state to either an overactivated or functionally inhibited phenotype, exacerbating pathology via aberrant cytokine release, dysregulated synaptic pruning and impaired myelin support. These changes are modulated by genetic susceptibility, sex differences, environmental stressors and microbiome alterations. We then survey translational advances—traditional and novel therapeutics that modulate microglial polarization, emerging blood- and imaging-based biomarkers, and strategies to harness microglia–oligodendrocyte cross-talk for remyelination—and highlight integrative platforms for stratifying inflammation-driven versus non-inflammatory subtypes. Our principal takeaway is that microglia represent a unifying nexus and actionable target for precision interventions tailored to individual biological profiles.

## 1 Introduction

**FIGURE 1:**
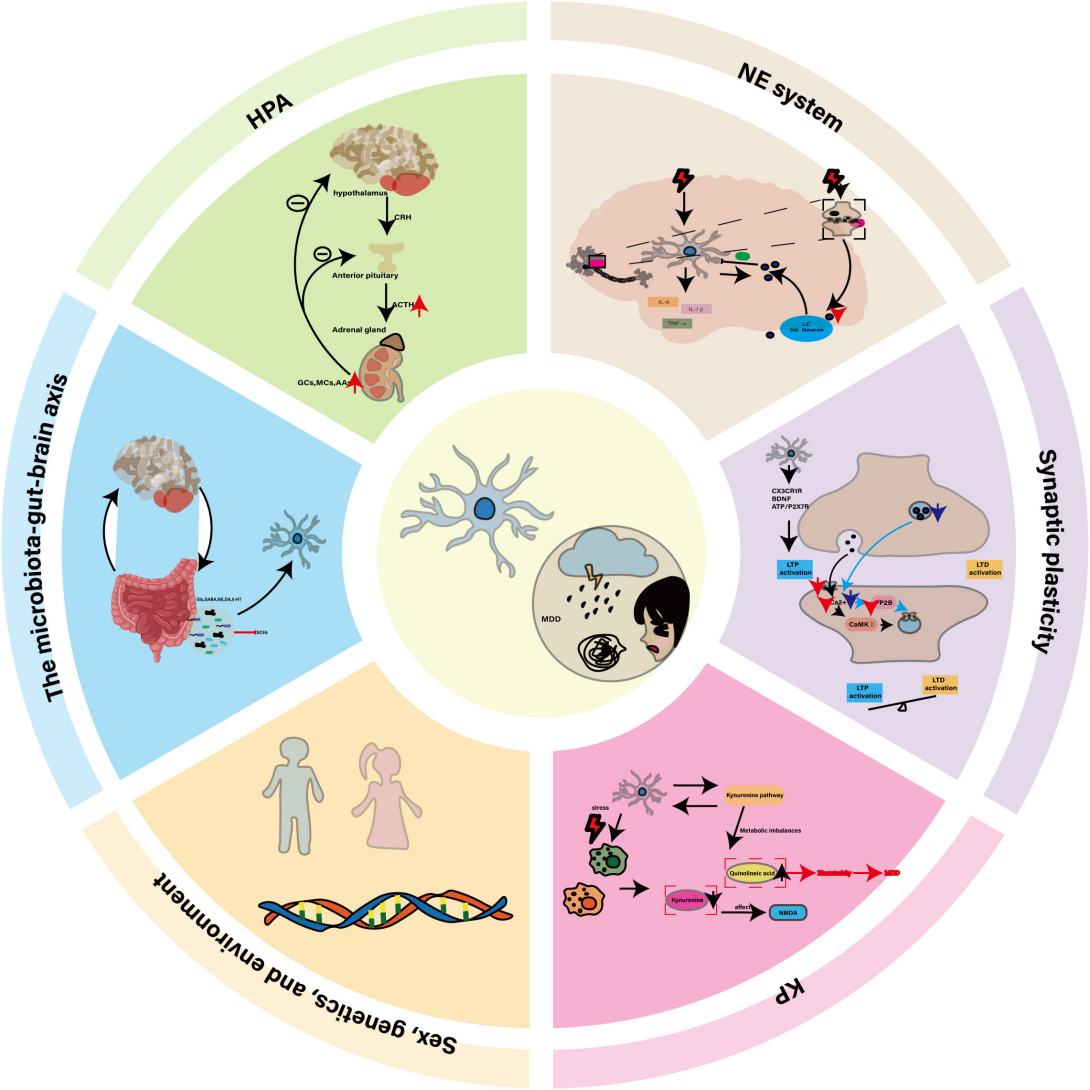
The central role of microglia in MDD and its potential as a therapeutic target. In MDD, dysregulation of the HPA axis, disturbances in the NE system, neuroinflammation, impaired synaptic plasticity, and KP imbalance collectively constitute the core pathological mechanisms. Microglia, as key regulators, interact with genetic factors, sex differences, environmental influences, and the gut-brain axis through their abnormal activation, release of inflammatory factors, and altered synaptic pruning functions, thereby driving the onset and progression of the disease.

Major depressive disorder (MDD) manifests as persistent low mood, anhedonia and loss of interest in previously enjoyable activities, and in severe cases may lead to suicidal behavior (Marx et al., 2023). Some patients also exhibit somatic changes such as weight loss, fatigue and diminished appetite. An estimated 300 million people worldwide suffer from MDD, making it a leading cause of disability whose prevalence continues to rise (Jin, 2016). Its etiology is multifactorial, encompassing dysregulation of the HPA axis, monoamine neurotransmitter imbalances, chronic inflammation, genetic and epigenetic factors, structural and functional brain remodeling, and psychosocial stressors (GBD 2017 Disease and Injury Incidence and Prevalence Collaborators, 2018). Although these hypotheses offer valuable insights, they each capture only part of the disorder’s complexity, and research has traditionally focused on neurons while overlooking the critical contributions of non-neuronal cells—especially microglia (Cui et al., 2024; Wang H. et al., 2022). As resident immune sentinels of the brain, microglia continuously survey the microenvironment, clear apoptotic debris and maintain homeostasis, Under pathological stress, they undergo rapid morphological and functional transitions, amplifying inflammation and disrupting neural networks (Kracht et al., 2020; Woodburn et al., 2021). In this review, we examine the central role of microglia in MDD pathogenesis and evaluate their potential as therapeutic targets (see Figure 1).

## 2 Microglia biology in health and MDD

### 2.1 Function and characteristics of microglia in healthy condition

Under normal physiological conditions, microglia exhibit a highly branched and active morphology, which is remarkably plastic and closely related to changes in cell function and brain signaling (Green and Rowe, 2024). Under resting conditions, microglia are evenly distributed in the brain parenchyma in fine branch shapes, like “sentinels” in the brain, playing an immune surveillance role. By continuously monitoring dynamic changes in the microenvironment and eliminating apoptotic neurons, it effectively maintains the homeostasis of the CNS and plays a fundamental role in ensuring the normal physiological function of the neural network. It is also known as the M0 phenotype of microglia. In the early stages of brain development, microglia finely shape and optimize the structure and function of the nervous system by regulating the number of neurons, participating in synaptic pruning, and affecting the formation and maturity of neural circuits (Borst et al., 2021). However, when brain homeostasis is unbalanced due to injury or infection, microglia can respond quickly, changing its form from a resting branched shape to a round or amoeba-like shape to enhance its phagocytic and clearance functions, promptly Clear damaged cells and potentially harmful substances and maintain the stability of the neural network (Nissen, 2017). However, under the excessive effect of specific external stimuli, microglia may become over-activated and release a large amount of inflammatory mediators, which in turn may lead to pathological phenomena such as excessive pruning of nerve synapses and neuron damage (Lewis, 2021).

In previous studies, the M1/M2 dichotomy has been widely used in the field of microglia, simply dividing the activation status of microglia into pro-inflammatory M1 type and anti-inflammatory M2 type (Kobashi et al., 2020). M1 microglia are usually activated by LPS and IFN-γ and express CD16, CD32, CD40, CD80 (B7-1), CD86 (B7-2) and MHC-II, while M2 microglia are activated by IL-4 and IL-13 and express CD68, CD206, Ym1 and Arg-1. This classification has provided a basis for researchers to initially analyze the response patterns and functional characteristics of microglia under different stimulation signals, but this dichotomy has limitations because it oversimplifies the real complex situation of microglia activation and is difficult to reflect the dynamic changes and transition states of cells in different environments. In fact, the activation state of microglia is not limited to M1 and M2 types, but rather conforms to the characteristics of a complex continuum. For example, M2 microglia can be further divided into three subtypes: M2a, M2b and M2c. M2a is the initial stage of microglia activation, which has anti-inflammatory properties; M2b is a mixed activation state, which can produce pro-inflammatory and anti-inflammatory cytokines; M2c has immunosuppressive and reparative properties, secreting anti-inflammatory factors to inhibit immune response, phagocytize apoptotic cells and fragments, and promote tissue repair and remodeling. Under different pathophysiological microenvironments, microglia can exhibit diverse mixed states with both pro-inflammatory and anti-inflammatory characteristics, or intermediate transition states between M1 and M2. This complex activation pattern is particularly common in CNS disease progression. To provide a concise overview of microglial activation states in MDD, Table 1 summarizes their primary stimuli, characteristic markers and functional roles.

**TABLE 1:** Microglial phenotypes, markers and functional roles in MDD.

Phenotype	Primary stimuli	Surface/ secreted markers	Functional role in MDD
M0	Homeostatic cues	P2Y12, TMEM119, CX3CR1	Immune surveillance; synaptic pruning during development
M1	LPS, IFN-γ, chronic stress	CD16, CD32, CD86, iNOS, IL-1β, TNF-α	Pro-inflammatory cytokine release; synaptic damage
M2	IL-4, IL-13, resolution	CD206, Arg-1, Ym1, IL-10, TGF-β	Anti-inflammatory signaling; phagocytic clearance; repair

Microglia phenotypes are thought to exist in a dynamic continuum, inspired by developments in single-cell transcriptomics and other high-resolution techniques, such as single-cell transcriptomics, which identified multiple functionally distinct microglia subsets in Alzheimer’s disease patients, including subsets of highly expressed antigen-presenting genes (CD74, H2-Aa), anti-inflammatory genes (IL-10, IL-4), and interferon-responsive genes (Bst2, Ifitm3) (Wang, 2021). Nguyen AT and his team took a single-cell transcriptome and found amyloid-reactive microglia-specific expression of CD163 (Nguyen et al., 2020). In addition, spatial transcriptomics revealed microglial dynamics around hippocampal plaques. The accumulation of microglia around plaques disrupts astrocyte signaling, leading to an imbalance in neuronal synaptic signaling, the study found (Mallach et al., 2024). The significant heterogeneity in cellular composition of plaque niche and the gene expression patterns of microglia in different plaque regions varied with distance further support the dynamic continuity of microglia phenotype, suggesting that their function and status may dynamically adjust with environmental changes.

### 2.2 Function and characteristics of microglia in MDD

Microglial dynamics combine neuroprotective and neurotoxic potential in MDD (Zhang and Tang, 2024). Microglia in the gray matter of the occipital cortex of patients with MDD often show an immunosuppressive phenotype, with reduced CD45 levels and down-regulated expression of genes such as CD163, Ki-67, osteopontin, CD14, CD64 and C1q chain. Animal models of chronic stress have shown that microglia have morphologic changes, with enlarged cell bodies and shortened processes, enhanced phagocytic activity in brain areas such as PFC, HIP and AMY, and increased CSF1 gene expression in the PFC. This phenomenon also occurs in the dorsolateral PFC of autopsies of MDD patients. However, genes associated with phagocytic activity are down-regulated in postmortem brain tissue from MDD patients, suggesting a possible decrease in phagocytic activity, whereas chronic stress models show an increase in phagocytic activity of microglia, suggesting that phagocytic function of microglia is complex in MDD.

Clinical evidence suggests that the anterior cingulate cortex (ACC) is abnormally connected to other brain regions in MDD patients (Zheng et al., 2018). Positron emission tomography (PET) reveals increased TSPO levels in ACC in patients with moderate to severe depression, indicating microglial activation, and higher TSPO levels in patients with suicidal ideation (Holmes et al., 2018). Increased microglial immune response in ACC subdomain of MDD patients correlates with increased density of quinoline-positive cells for the NMDAR agonist via kynurenine pathway (KP), providing new evidence for the glutamatergic dysregulation hypothesis of MDD (Steiner et al., 2011). Torres-Platas et al. (2014) also found an increased proportion of resting microglia in the dorsal ACC white matter and more than twice the density of perivascular macrophages in middle-aged MDD suicides, consistent with the neuroinflammatory theory of MDD. Taken together, these clinical evidences reveal a potential link between microglial activation and the onset of MDD. Animal models can help to further understand the link between inflammation and MDD and outline the abnormal activation of microglial cells under stress conditions.

**FIGURE 2:**
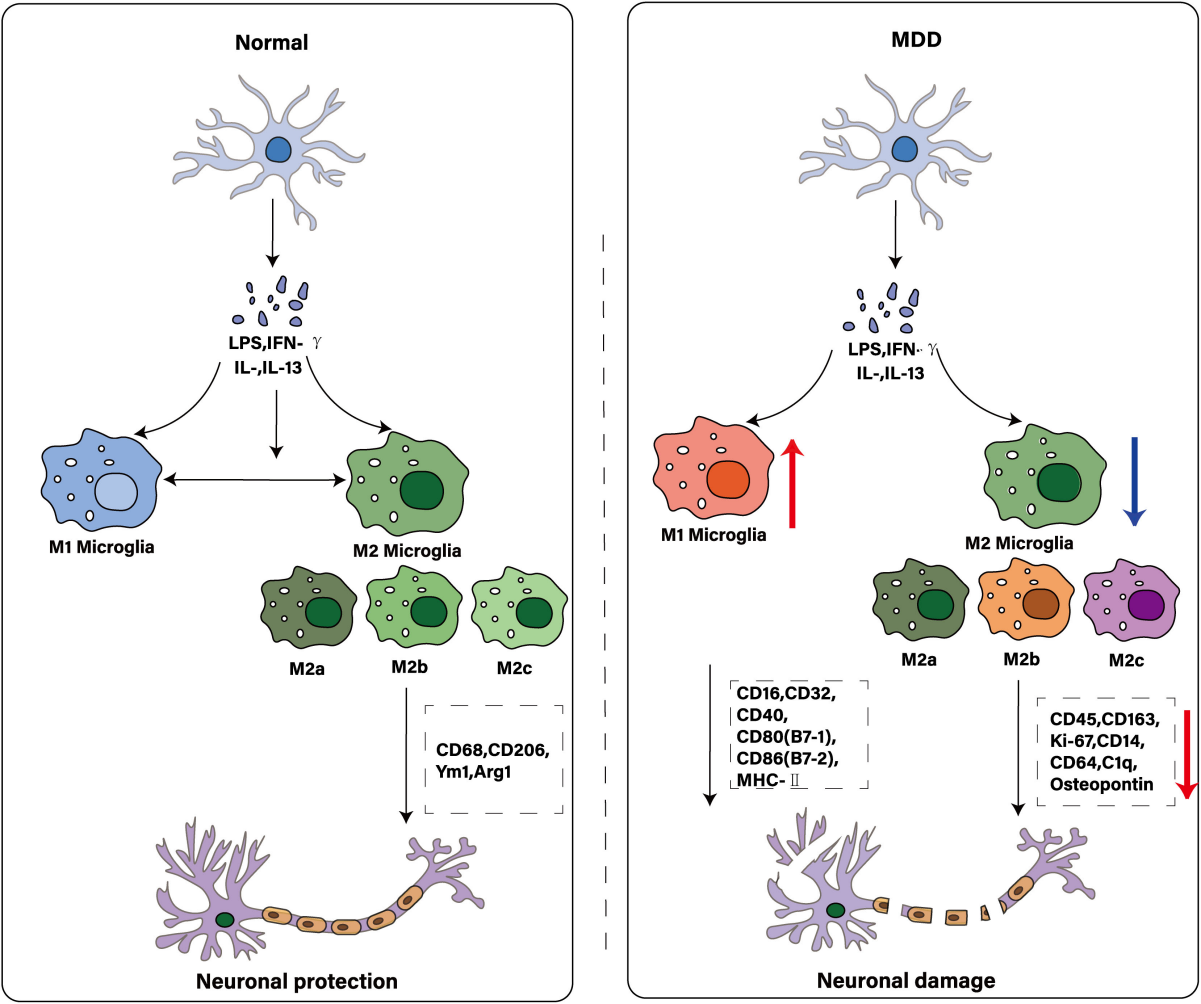
Comparison of microglial polarization states: normal and MDD. The illustration depicts microglia as key immune cells in the central nervous system. While traditionally characterized by M1 and M2 polarization states, these cells exhibit greater diversity - including multiple M2 subtypes (e.g., M2a, M2b, M2c)–each with distinct activating factors and functions. Under physiological conditions, microglia maintain homeostasis to protect neurons. In MDD, this equilibrium may be disrupted: increased M1 polarization promotes neuronal damage, while reduced M2 activity diminishes immunosuppressive and neuroprotective functions.

Microglia are involved in immune responses through Toll-like receptors (TLRs). TLR3 and TLR4 can be activated by specific ligands, such as polyinosine-polycytidine [poly(I:C)], which activates TLR3, and lipopolysaccharide (LPS), which activates TLR4. The simultaneous activation of both induces the release of proinflammatory cytokines and triggers morphological and functional changes in microglia in CNS, which enhances the inflammatory response of microglia (Pišlar et al., 2022). Animal models of multiple forms of stress exposure, similar to psychosocial stress in MDD patients, can induce depression-like behavior and microglial activation, and are unique sites for studying the role of microglia in the pathogenesis of MDD. To this end, researchers have developed multiple animal models of MDD, such as the chronic unpredictable stress (CUMS) model (Liu et al., 2025), the chronic mild stress (CMS) model (Liu et al., 2022), the chronic social frustration stress (CSDS) model (Li B. et al., 2022), the maternal deprivation (MD) model, and the prenatal stress model (Zheng et al., 2024). By monitoring behavioral endpoints such as weight, appetite, psychomotor activity, sleep, anxious behavior, social interaction, despair and anhedonia, as well as assessing cognitive dysfunction, levels of oxidative stress, levels of monoamine neurotransmitters and BDNF, and HPA axis activity. Biomarkers can comprehensively assess the depression-like symptoms of these models, thereby providing information support for the study of the pathological mechanism of MDD and the development of new treatment methods. As shown in Figure 2.

## 3 Pathophysiological mechanism

### 3.1 HPA axis

The hypothalamic–pituitary–adrenal (HPA) axis constitutes the body’s principal stress-response system, coordinating endocrine, immune and behavioral adaptations. Under acute challenge, corticotropin-releasing hormone (CRH) from the hypothalamus drives adrenocorticotropic hormone (ACTH) release, which in turn spurs glucocorticoid (GC) secretion from the adrenal cortex. Glucocorticoids—principally cortisol in humans—engage glucocorticoid receptors (GRs) in multiple cell types, including microglia, to terminate the stress response via negative feedback (Du et al., 2023; Fan et al., 2025; Peles et al., 2023).

In major depressive disorder (MDD), this feedback loop is dysregulated: many patients exhibit chronically elevated cortisol and impaired GR sensitivity (Bertollo et al., 2025; Chaves et al., 2025). Pharmacological dampening of HPA hyperactivity—whether by GR antagonists or by inhibitors of stress-responsive kinases—reduces depressive-like behavior in rodents (Luhong et al., 2024). For instance, ISRIB treatment in chronic restraint stress (CRS) models lowers serum corticosterone and hippocampal GR expression, restoring sucrose preference and reducing immobility in forced-swim tests (Xie et al., 2025). Likewise, maternal corticosterone exposure disrupts offspring hippocampal BDNF-mTOR signaling, elevates ACTH/CRH and impairs neurogenesis, mirroring features of postpartum depression (Pandey et al., 2013). Postmortem analyses further reveal decreased GR-related gene expression in the prefrontal cortex and amygdala of adolescent suicide victims (Sierra et al., 2008), implicating defective HPA feedback in human MDD.

**FIGURE 3:**
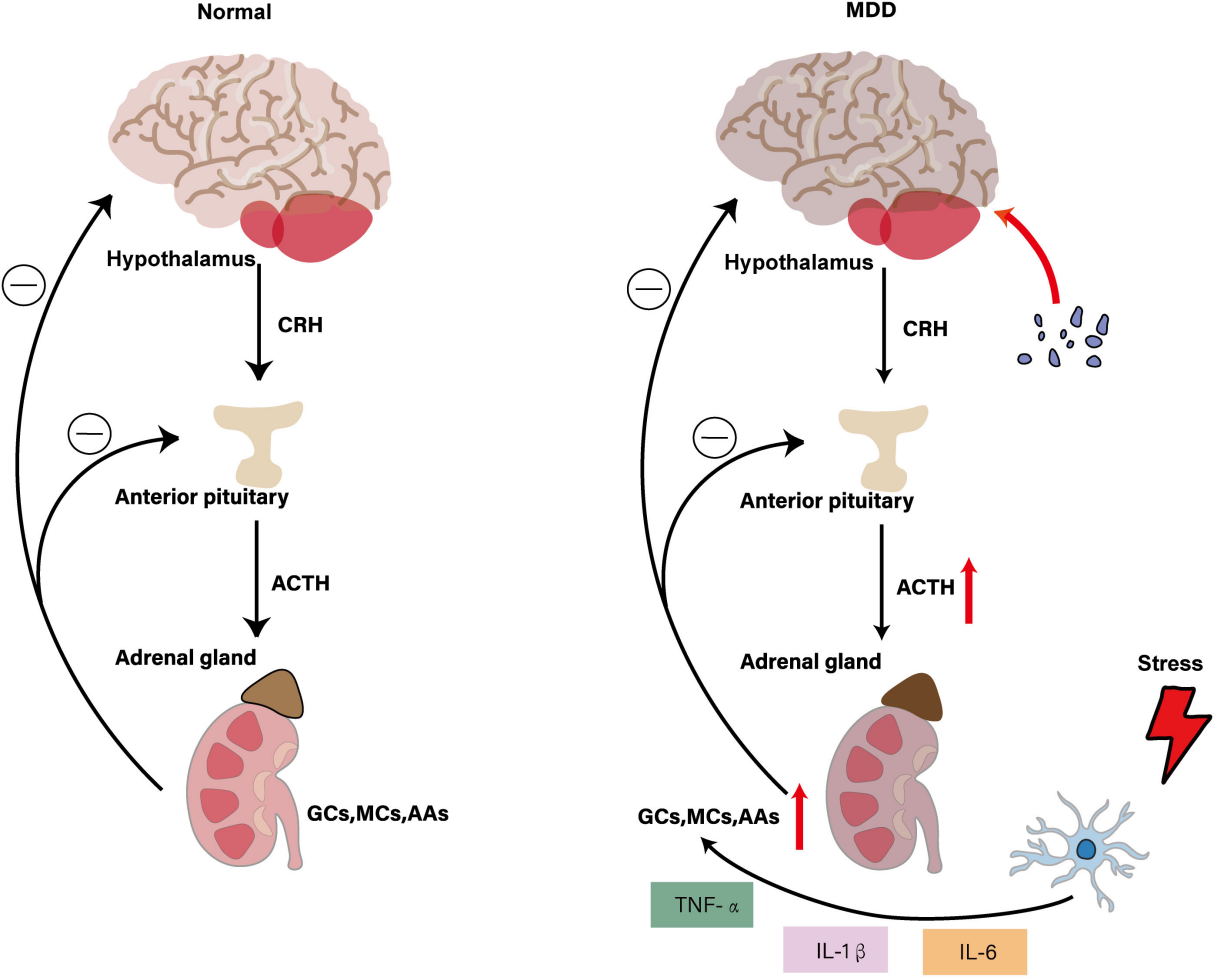
Interaction of HPA axis dysregulation with microglial activation in MDD. The figure illustrates that under normal physiological conditions, the hypothalamus secretes CRH to stimulate ACTH release from the anterior pituitary, which subsequently promotes adrenal secretion of GCs, MCs and AAs. These hormones maintain HPA axis homeostasis through negative feedback mechanisms. In the MDD state, HPA axis hyperactivity leads to increased CRH and ACTH secretion, resulting in elevated adrenal hormone production. Stress-activated microglia release pro-inflammatory cytokines (TNF-α, IL-1β and IL-6), which further stimulate the HPA axis, creating a vicious cycle. This leads to pathological microglial activation and enhanced neuroinflammation, ultimately affecting mood regulation and behavior while promoting the development of depressive symptoms.

Microglia themselves express GRs and respond directly to GC fluctuations (Singh et al., 2024). Under chronic stress, GC-driven GR activation paradoxically “primes” microglia—shifting them from a homeostatic to a pro-inflammatory state. In restraint stress paradigms, pre-treatment with GR antagonists blocks stress-induced microglial proliferation (Feng et al., 2019; Kim et al., 2019; Nair and Bonneau, 2006), while excessive GC dosing triggers NF-κB–NLRP3 pathway activation in hippocampal microglia, driving IL-1β and IL-6 release and precipitating depressive-like behaviors (Sorrells et al., 2009). These cytokines further stimulate CRH and ACTH secretion, creating a feed-forward loop that entrenches neuroinflammation and HPA dysregulation. Most mechanistic insights stem from rodent CRS or acute restraint models, which differ markedly in duration and psychosocial complexity from human depression (Park et al., 2020; Santos et al., 2016; Zhao et al., 2017). *In vivo* PET imaging of GR occupancy in MDD patients suffers from low signal-to-noise and lacks ligands that distinguish microglial from neuronal GR (Slavova et al., 2024). Postmortem studies are cross-sectional and confounded by medication history and agonal factors. To overcome these hurdles, we recommend longitudinal cohort studies that integrate single-cell GR reporters, conditional GR knockouts in hippocampus versus prefrontal cortex, peripheral cortisol profiling and high-resolution *in vivo* imaging of microglial calcium signaling. Moreover, emerging evidence links HPA-axis–driven microglial priming to peripheral immune and metabolic pathways. GC-activated microglia disrupt gut-barrier integrity and enhance indoleamine-2,3-dioxygenase (IDO) activity, redirecting tryptophan toward neurotoxic quinolinic acid rather than kynurenic acid. This positions HPA dysregulation as a central hub that interconnects stress, systemic inflammation and kynurenine pathway imbalances via microglial crosstalk (Figure 3). These HPA-axis-driven microglial changes also compromise gut-barrier integrity and peripheral immune tone, thereby predisposing to gut–brain axis disturbances and heightened neuroinflammation.

### 3.2 NE system

NE system plays an important regulatory role in central nervous system. Its core structure, locus coeruleus (LC) neurons, widely project to many brain regions and participate in the regulation of physiological functions such as emotion, arousal, cognition and stress response by acting on adrenergic receptors (Wu and Baeken, 2024). NE system dysfunction is one of the core biological characteristics of MDD, which is characterized by decreased activity of LC neurons, decreased NE synthesis and release, and further affects mood regulation, arousal maintenance, cognitive function and stress response, resulting in depression and inattention (Cornell et al., 2022). Microglia, as the core immune cells of the central nervous system, are over-activated in MDD, releasing pro-inflammatory cytokines to trigger neuroinflammation, and aggravating neuronal damage and synaptic plasticity through abnormal synaptic pruning and blood-brain barrier destruction (Lechtenberg et al., 2019). NE system and microglia have bidirectional regulation: NE inhibits microglia activation through β2 adrenergic receptor (AR) and reduces pro-inflammatory factor release (Hou et al., 2021); microglia-mediated neuroinflammation interferes with NE neuron function through cytokines (Stowell et al., 2019), forming a vicious circle of “neuroinflammation-transmitter imbalance.”

NE system plays an important role in regulating microglial activity. NE neurons in LC are active during wakefulness and inactive during sleep. This rhythmic change regulates microglial surveillance function and synaptic plasticity (Anderzhanova et al., 2020). Under stress conditions, such as maze exploration and physical restriction, LC neuron activity can be enhanced and NE release can be promoted (Morris et al., 2020). Chronic stress not only increases LC neuron responsiveness to subsequent stress, but also may aggravate depressive symptoms through interaction with CRH system (Wang et al., 2017). α2A-AR is the main inhibitory autoreceptor of NE neurons, which is involved in the regulation of NE release. It is found that chronic stress can significantly enhance α2A-AR mediated presynaptic inhibition, reduce NE secretion from LC to paraventricular nucleus of hypothalamus, and block hyperactivated α2A-AR can increase NE level in paraventricular nucleus of hypothalamus and improve depressive symptoms (Sugama et al., 2019). In addition, stress-induced microglial activation is mainly mediated by β1-AR and β2-AR, and NE is involved as a key neurotransmitter. Under acute stress, microglial cells show morphological activation such as increased cell size and increased expression of Iba1, which can be significantly inhibited by β-blockers. In β1-and β2-AR deficient double knockout mice, stress-induced microglial morphological changes are abolished, indicating that NE system is involved in the development of depression by regulating microglial activity (Maggi et al., 1980). In the central nervous system, activation of β-AR increases α2-AR binding sensitivity, and this modulation may be involved in maintaining homeostatic control of NE activity (Li et al., 2019). This internal regulatory mechanism further illustrates how the NE system maintains functional homeostasis through interactions between its own receptors, thereby affecting the pathological process of MDD.

**FIGURE 4:**
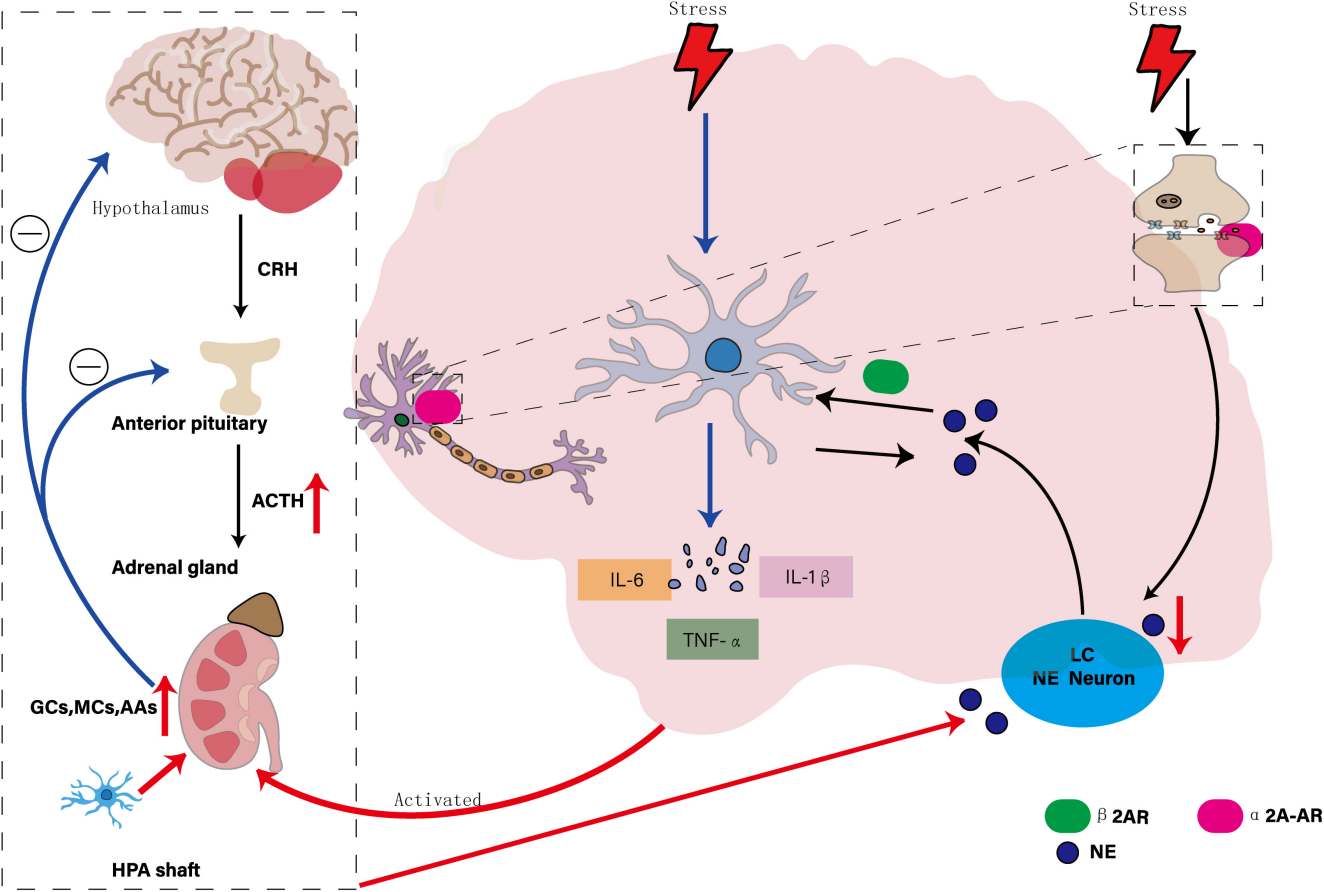
Interaction of the NE system, microglia, and MDD. The diagram illustrates the bidirectional regulatory mechanisms between the NE system and microglia: NE suppresses microglial activation by stimulating the β2-adrenergic receptor (β2AR), thereby reducing the release of pro-inflammatory factors. Conversely, microglia-mediated neuroinflammation disrupts the function of NE neurons through the release of cytokines. Chronic stress significantly enhances α2A-AR-mediated presynaptic inhibition, leading to reduced NE secretion from the locus coeruleus (LC) to the paraventricular nucleus of the hypothalamus. The overactivation of pro-inflammatory cytokines further stimulates the HPA axis, increasing the secretion of stress hormones such as glucocorticoids (GCs), which in turn inhibit the synthesis and release of NE, affecting its availability in the synaptic cleft.

In the context of MDD, chronic stress activates microglia, prompting them to release a variety of pro-inflammatory cytokines (e.g., IL-1β, TNF-α, IL-6) that affect NE systems through a variety of mechanisms. On the one hand, pro-inflammatory cytokines overactivate HPA axis, which leads to increased secretion of stress hormones such as GCs, and then inhibits NE synthesis and release, reduces NE concentration in synaptic gap, affects signal transmission between neurons, and reduces NE availability. Proinflammatory cytokines, on the other hand, increase tryptophan 2,3-dioxygenase activity, promote the conversion of tryptophan to kynurenine, reduce serotonin (5-HT) synthesis, and indirectly affect NE system function (Kitayama et al., 2008). Animal model studies showed that NE neurons in LC degenerated and microglia increased significantly in the rat model of MDD, suggesting that NE neuron degeneration and microglia responses may be involved in the pathological process of MDD together (Pozzo Tremolanti et al., 2019). In conclusion, NE system dysfunction not only affects neurotransmitter balance, but also triggers inflammatory response and neurodegenerative changes by regulating microglia activity, aggravating depressive symptoms, suggesting that regulating NE system or inhibiting microglia overactivation may be potential strategies for treating MDD, as shown in Figure 4.

Despite extensive rodent work implicating β-adrenergic signaling in microglial activation, most data derive from germline knockouts or pharmacological blockade without temporal specificity, making it difficult to parse developmental from adult-onset effects. *In vivo* microdialysis studies linking NE fluctuations to microglial cytokine release typically involve small sample sizes (*n* < 8) and are limited to striatal or hippocampal sites, leaving cortical noradrenergic control underexplored. Human imaging of the locus coeruleus remains technically challenging due to its small size and proximity to CSF, and current neuromelanin MRI protocols lack cell-type resolution. Future work should employ inducible, region-specific β-AR manipulations in mammals and leverage high-field MRI coupled with novel chemogenetic sensors to map NE–microglia interactions in humans.

### 3.3 Inflammatory environment

Inflammation is a defense response initiated when immune cells are activated to clear pathogens, toxins, and tissue damage. Inflammatory environments are composed of inflammatory cells, mediators, and signaling pathways that are critical to maintaining homeostasis. When the inflammatory environment is disturbed by external stimuli, the predominance of pro-inflammatory signals will trigger acute inflammation, which will easily develop into chronic inflammation if it is continuously activated and not balanced by anti-inflammatory mechanisms. In CNS, neuroinflammation can destroy neuronal function, affect neurotransmitter balance and synaptic transmission.

The pathophysiological process of depression is closely related to inflammatory environment. Activation of inflammatory pathway in brain can trigger excitotoxicity and oxidative stress, which can lead to neuronal damage and cognitive and executive function changes. MDD patients are often accompanied by increased hormone release, plasma pro-inflammatory cytokine levels and oxidative stress indicators (Ruiz et al., 2022). Hashimoto O and his team found that depressed behaviors induced by early life stress exacerbate the adolescent brain’s susceptibility to systemic inflammation. Proinflammatory cytokines activate the microglial tryptophan kynurenine pathway, up-regulating key rate-limiting enzymes of the pathway in the hippocampus, prefrontal cortex and amygdala, and increasing the density of Iba-1-positive microglia, resulting in a decrease in the volume and thickness of these brain regions (Hashimoto et al., 2020). Functional magnetic resonance imaging revealed differences in the functional connections of the resting default mode network and ventral attention network with peripheral inflammatory markers IL-6 and TNF-α between MDD patients and healthy subjects, further suggesting that peripheral inflammation is associated with MDD status (Schmitz et al., 2025). Genome-wide association studies and epigenetic analysis revealed that genetic variants associated with depression were significantly enriched in epigenetic active sites of activated CD4+T lymphocytes, which may affect immune cell function, promote the increase of peripheral blood inflammatory markers (CRP, IL-1β, TNF-α, IL-6, IL-17) and increase leukocyte count, suggesting that the immune system may participate in the pathogenesis of MDD through inflammatory response (Lynall et al., 2022).

Microglia, as immune cells of CNS, play a central role in inflammatory environment. In chronic stress model, microglia can induce anxiety and depression by reducing the expression of NKAα1 on microglia membrane, increasing free P2 × 7R, triggering potassium outflow, activating NLRP3 inflammatory signal, releasing IL-1β and other factors; inhibiting microglia inflammatory response can reverse neuroregeneration disorder and anxiety-like behavior (Huang et al., 2024). The imbalance of pro-inflammatory and anti-inflammatory factors in microglia contributes to the development of MDD. In MDD patients, microglia often exhibit M1 overactivation and M2 underfunction, resulting in increased pro-inflammatory factor release and decreased anti-inflammatory factor release, triggering neuroinflammation, disrupting neuronal function and synaptic plasticity, thereby exacerbating depressive symptoms. the study found that TSPO regulate microglia phenotype, activation of TSPO increases that release of anti-inflammatory cytokine IL-4 and IL-10, decreases the release of pro-inflammatory cytokines IL-6 and IL-8, and reduces neuroinflammation (Liu Y. et al., 2024).

Regulating the M1/M2 balance of microglia is one of the important targets for improving the inflammatory environment and treating MDD. Curcumol regulates Treg/Th17 balance and reduces neuroinflammation by promoting the transition of microglia from M1 to M2 (Liu L. et al., 2024). Running can also regulate microglia phenotype by increasing adiponectin levels and activating AdipoR1 receptors, alleviating neuroinflammation and depressive symptoms caused by CUMS (Li W. et al., 2022). Adiponectin knockout mice exhibit antidepressant behavior in LPS-induced MDD models, which may be related to the interaction of TrkB/BDNF and NFκB signaling pathways (Li et al., 2024). In addition, deletion of Pdcd4 reduces LPS-induced overactivation and depression-like behavior of microglial cells, and increases expression of the anti-inflammatory factor IL-10 through Daxx-mediated PPARγ nuclear transfer (Reichert Plaska et al., 2024). Ginsenoside Rb1 activates PPARγ to induce microglia to express M2-like phenotype and reduces depression-like behavior (Ślusarczyk et al., 2018). Azurin inhibits the polarization of microglia toward the M1 phenotype and reduces neuroinflammation by inhibiting the NLRP3 inflammatory body and regulating TLR4, ERK1/2, and NF-κB signaling pathways (Liu B. et al., 2021). DPA can promote the polarization transition of microglia from pro-inflammatory M1 to anti-inflammatory M2 by inhibiting the NF-κB/MAPKp38 signaling pathway, and exert anti-neuroinflammatory and neuroprotective effects by activating the BDNF/TrkB-PI3K/AKT signaling pathway in neurons (Rial et al., 2015). However, MDD is a heterogeneous disease, and although inflammation is the core pathogenesis of some patients, it is not the only one. Inflammation may be a core feature of some patients, but it lacks precise diagnostic criteria and biomarkers. Various treatments play an antidepressant role by regulating the M1/M2 balance of microglia and improving the inflammatory environment, but the direct correlation between these therapies and microglia is unclear, and the intermediary is the inflammatory environment itself. Intervening only inflammation may delay the development of MDD, which makes the specific mechanism of action of microglia unclear and clinical evidence is limited. At the same time, the specificity and sensitivity of existing inflammatory markers are insufficient, making it difficult to comprehensively reflect the role of microglia in inflammatory states and disease complexity. Future research needs to deeply explore the mechanism of action of microglia on specific inflammatory factors, explore more inflammatory markers, and provide a basis for the development of new clinical drugs for MDD. These inflammatory signals not only disrupt synaptic plasticity but also reshape the gut–brain dialog and shift kynurenine metabolites toward neurotoxic quinolinic acid, underscoring a tightly coupled axis of neuroendocrine, immune and metabolic dysregulation. This pro-inflammatory milieu not only disrupts synaptic integrity but also diverts tryptophan metabolism toward neurotoxic quinolinic acid, underscoring the coupling of immune and metabolic pathways in microglia-mediated MDD.

### 3.4 Synaptic plasticity

Synapses are connection structures for information transmission between neurons. They consist of pre-synaptic membranes, gaps and post-synaptic membranes, where neurotransmitters transmit signals. Synaptic plasticity refers to the dynamic adjustment of synaptic strength and function, which is related to learning, memory and neural network adaptability. LTP and LTD are two main manifestations of synaptic plasticity. In healthy conditions, LTP is a long-term enhancement of synaptic transmission efficiency. It is manifested in the release of a large amount of glutamate by presynaptic neurons when they are active, activating NMDA receptors in the postsynaptic membrane, depolarization removes Mg^2+^ blocking, and Ca^2+^ inflow activates CaMKII and cAMP/PKA pathways, promotes AMPA receptor insertion, and enhances synaptic strength. It is a key mechanism for learning and memory (Finley, 2018). LTD is a long-term decrease in synaptic transmission efficiency, manifested by decreased presynaptic neuronal activity, reduced glutamate release, reduced Ca^2+^ influx, activation of phosphatase PP2B, dephosphorylation and endocytosis of AMPA receptors, and decreased synaptic strength, which helps to optimize the coordination of neural networks and can improve cognitive flexibility and adaptability (Hell, 2016).

Recent studies have found that MDD is closely related to impaired synaptic plasticity (Zhvania et al., 2024). In the hippocampus and prefrontal cortex of patients with MDD, the number of synapses is reduced, and glutamate release from presynaptic neurons is reduced, resulting in insufficient activation of NMDA receptors in the postsynaptic membrane, reduced Ca^2+^ influx, and limited activation of downstream signaling pathways. At the same time, PP2B activation decreased, affecting AMPA receptor dephosphorylation and endocytosis, inhibiting LTP formation and enhancing LTD, thus affecting learning, memory and information transmission (Wei et al., 2002). In the CUMS model, the ratio of precursor (proBDNF) to mature (mBDNF) of brain-derived neurotrophic factor (BDNF) was up-regulated. Since BDNF is a key upstream regulator of LTP, changes in this ratio lead to disorders of LTP, which in turn leads to cognitive decline and reduces synaptic plasticity in the hippocampus (Zhang et al., 2016). In the chronic stress model, the number of axon-spinous synapses and myelinated axons in the deep cortical layer (layers III-VI) of the medial prefrontal cortex (mPFC) of rats decreased, the length of synaptic membrane increased, and the connectivity of the neuronal network decreased. These changes may be related to stress-induced dendritic atrophy and reduced spinous process density, and may be a compensatory adjustment for the reduced number of synapses (Csabai et al., 2018).

The dynamic interaction of microglia with synapses plays a key driving role in synaptic plasticity (Kleidonas et al., 2023), affecting the stability and adaptability of neural circuits, which in turn affects emotional and cognitive function (Innes et al., 2019). It regulates synaptic plasticity through pathways such as the CX3CR1 receptor, inflammatory factors (IL-1β, TNF-α), BDNF and ATP/P2 × 7 receptor, and plays a role in the pathological process of MDD (Hei et al., 2019). In the CMS model, activation of microglia led to increased expression of spinophilin in the postsynaptic dense layer protein in the hippocampus, reduced the density of synaptic vesicles in the inner and outer layers of the molecule, increased the number of spines in the inner and outer layers of the molecule, and compensated changes in the synaptic structure, which in turn impaired spatial memory and exploration ability (Cheng et al., 2024). In the CUMS model, microglia can attenuate the release of inflammatory factors by blocking the CX3CR1-CX3CL1 pathway, enhance adaptability to excessive permeability of the blood-brain barrier and loss of dendritic spines, increase synaptic plasticity, reduce depression-like behaviors such as anhedonia, anxiety or despair, and improve cognitive impairment (Liu et al., 2020). Other studies have found that 10 Hz repeated transcranial magnetic stimulation can trigger microglia to release TNFα and IL-6, which in turn promotes synaptic plasticity (Xiao et al., 2022). In the CUMS model, MST1 activation impairs hippocampal synaptic plasticity and abnormally activates microglia, increasing neuroinflammation; while reducing MST1 levels reduces microglia activation, suppresses inflammatory responses, and promotes synaptic plasticity through the BDNF/AKT/CREB signaling pathway to counter depressive behavior and cognitive impairment caused by CUMS (Xu et al., 2021). In the CNS, P2 × 7R is an ATP-gated cation channel located in different cell types. P2 × 7R activation in microglia regulates the innate immune response through inflammatory bodies. Studies have found that P2 × 7 is up-regulated in the cortex of patients with frontotemporal lobe degeneration. Further research has shown that P2 × 7 deficiency can significantly reduce microglia activation and inhibit the inflammatory mediator CCL4, thereby improving synaptic plasticity and memory impairment (Ribeiro et al., 2019).

**FIGURE 5:**
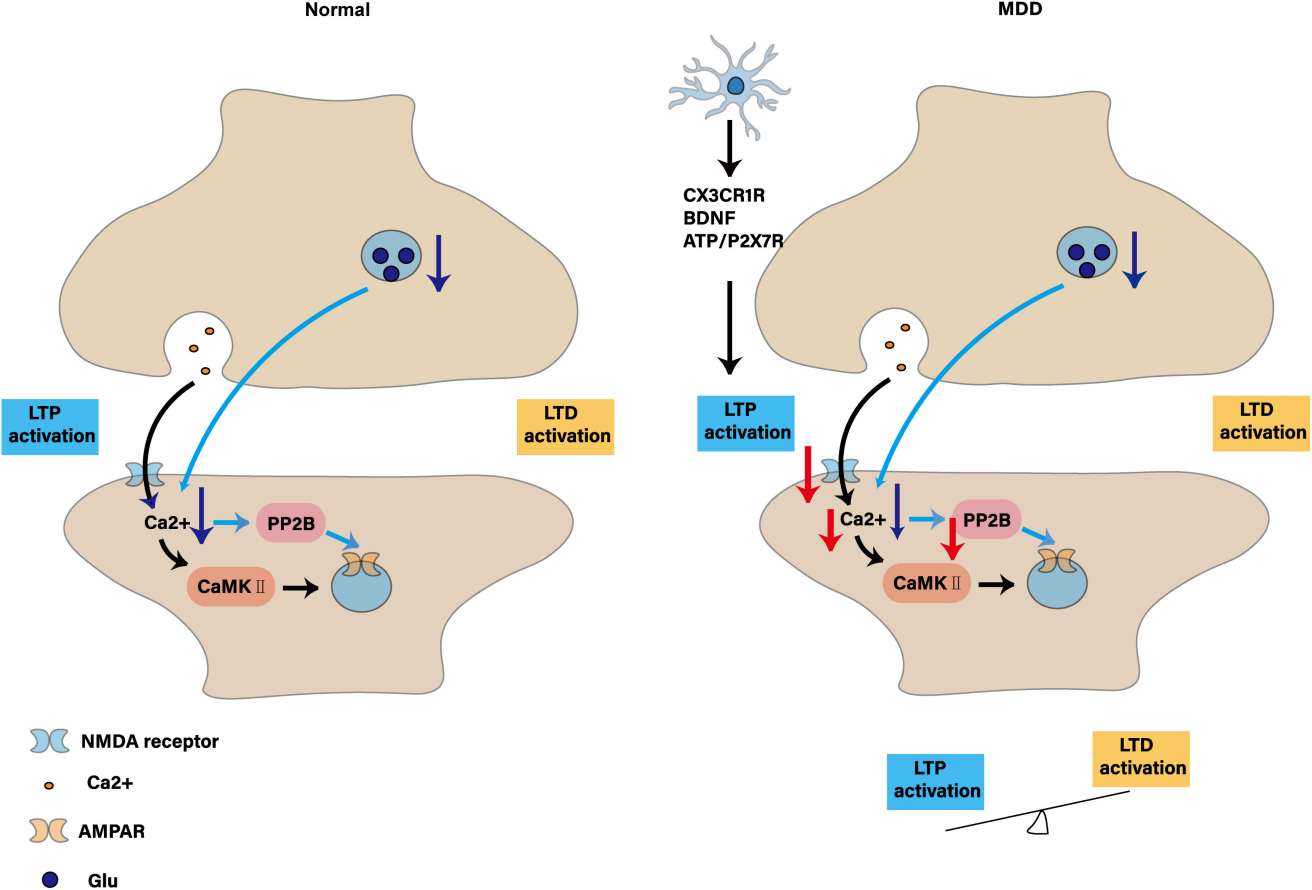
Synaptic plasticity: contrasts in physiology and depression and the regulatory role of microglia. The figure shows synaptic plasticity under normal conditions: LTP activation: presynaptic neurons release Glu, which activates NMDA receptors on the postsynaptic membrane, resulting in Ca^2+^ influx, which in turn activates the protein kinases CaMK II and PP2B, promotes phosphorylation and insertion of AMPA receptors, and enhances synaptic transmission efficiency. LTD activation: decreased presynaptic neuronal activity, decreased Glu release, decreased Ca^2+^ influx, activation of PP2B, leading to dephosphorylation and endocytosis of AMPA receptors, and attenuation of synaptic transmission efficiency. Synaptic plasticity in MDD: impaired LTP activation: microglia release inflammatory factors through CX3CR1R, BDNF, ATP/P2X7R and other pathways, interfere with the activation of NMDA receptors in the postsynaptic membrane, reduce Ca^2+^ influx, affect the activation of CaMK II and PP2B, hinder the insertion of AMPA receptors, and lead to LTP formation disorders. LTD enhancement: due to the impaired LTP, the synaptic transmission efficiency is weakened, resulting in the synapse being “pruned,” and the LTD and LTP are “out of balance,” making it difficult to form new connections, affecting learning and memory and information transmission.

Microglia are a key target for improving depression and regulating synaptic plasticity, and are more effective in patients with early MDD. In patients with MDD or bipolar disorder in the middle and late stages, due to CNS synaptic damage and plastic destruction, it is not enough to increase monoamine neurotransmitters in the synaptic gap, and the neural network needs to be repaired. Whether microglia are the only target is uncertain, and most studies use multi-target strategies to improve synaptic plasticity and depression-like behavior. It will be of great value to clarify the proportion of microglia contributing to synaptic plasticity in the future. However, improving synaptic plasticity, repairing neural networks and maintaining their homeostasis remain the key to treating MDD. As shown in Figure 5.

Rodent electrophysiological assays of LTP and LTD provide valuable mechanistic insight but do not capture the complexity of human cortical synapses, which exhibit distinct NMDA-to-AMPA ratios and plasticity thresholds. Current synaptic markers (e.g., PSD-95, synaptophysin) lack the spatial and cell-type specificity needed to resolve microglia–neuron interactions *in situ*. Moreover, longitudinal imaging of synaptic changes *in vivo* has been restricted to small animal models and cannot yet be translated to clinical cohorts. Moving forward, the field should validate PET tracers for SV2A alongside two-photon microscopy in non-human primates and develop genetically encoded fluorescent reporters compatible with human cortical organoids to bridge this translational divide.

### 3.5 KP

Building on stress- and cytokine-driven signals as well as gut-derived modulators, microglial induction of indoleamine-2,3-dioxygenase (IDO) serves as a convergent node that integrates HPA, inflammatory and gut–brain inputs into kynurenine pathway alterations. Tryptophan is an essential amino acid for the human body. As a precursor of KP, it is converted to kynurenine (KYN) under the action of the rate-limiting enzymes IDO and TDO. KYN is catalyzed by KAT to produce KYNA. KYNA is an endogenous NMDA receptor antagonist that can scavenge free radicals and exert a neuroprotective effect (Santamaría et al., 1996). KYN produces 3-HK under the action of KMO, which is then converted into 3-HAA by KYN enzyme, oxidized into ACMS by 3-HAO, and further metabolized into QUIN. QUIN is an NMDA receptor agonist and can cause neurotoxicity (Castellano-Gonzalez et al., 2019). In addition, KYN can also be converted to anthranilic acid under the catalysis of KYN enzyme, which in turn produces NAD+. In the CNS, KYNA produced by KP increases synaptogenesis and neural plasticity by blocking the glycine B site on the NMDA receptor, countering the excitotoxicity caused by QUIN (Newell et al., 1997). QUIN binds to the NMDA receptor to increase intracellular Ca^2+^ concentration, causing excitotoxic damage, prompting neurons to release glutamate, inhibiting the reuptake of glutamate by glial cells, increasing extracellular glutamate levels, and further activating the glutamatergic system, leading to neuron death.

KP is closely related to MDD. Patients with MDD have abnormal levels of KP metabolites, increased neurotoxic product QUIN, and decreased neuroprotective product KYNA. Increased release of pro-inflammatory cytokines activates IDO and KMO, causes KP to produce more neurotoxic products such as QUIN, interferes with the dynamic balance of glutamate-glutamine-γ-aminobutyric acid, exacerbates glutamate excitotoxicity, and leads to neuron damage (Wu X. et al., 2022). A meta-analysis of 101 studies involving 10,912 participants found that tryptophan metabolism in patients with MDD shifted to KP, resulting in a decrease in tryptophan and KYN levels, a decrease in the ratio of KYNA to QUIN and the ratio of KYNA to 3-HK, and an increase in the ratio of KYN/tryptophan, indicating that abnormal changes in KP may be involved in the development of MDD (Marx et al., 2021). In the CUMS model, after 3–4 weeks, 5-HT levels in the hippocampus of mice decreased, KP transferred to the KYN-QA branch, resulting in a decrease in neuroprotective substances KYNA, accumulation of neurotoxic substances such as 3-HK and QA, and the mice developed anxiety-like behavior, manifested by a decrease in the ratio of time to open arms in the elevated cross maze, a prolonged eating latency in the novel inhibition feeding test, and a decrease in the number of light-dark box switches (Luo et al., 2025). After 4 weeks of CUMS exposure, the mRNA expression and enzyme activity of the KP rate-limiting enzyme IDO were up-regulated, the transcription of downstream KMO and KYNU genes increased, while sucrose preference decreased, and the performance of the Barnes maze test deteriorated, indicating the emergence of depressive behavior (Wang B. et al., 2018).

Abnormal activation of microglia can cause an imbalance in KP metabolism, which in turn produces neurotoxic substances, which is closely related to MDD. As a precursor of quinolinic acid in microglia, KYN activates NMDAR to increase glutamate release, which is associated with increased IDO activity, quinolinic acid and glutamate levels in the hippocampus, prefrontal cortex and serum. In the CUMS model, IDO inhibitor intervention inhibited microglia activation, regulated KP metabolism and glutamate content, inhibited the conversion of tryptophan to KYN, and improved depressive behavior in sucrose preference, tail suspension, and forced swimming tests (Bansal et al., 2022). In CUMS and LPS models, KXS and ginsenoside Rg3 reduced neuroinflammation by inhibiting the proliferation of hippocampal microglia, decreased IDO1, TDO2 and KMO, and restored peripheral and hippocampal tryptophan-KYN metabolism, manifested by increased levels of KYN, KYNA, KYN/tryptophan and KYNA/quinolinic acid, and decreased 5-HT concentrations, thereby improving depressive behavior in mice (Yao et al., 2025). Brooks et al. found that dexamethasone inhibits LPS-induced microglia activation and IDO1 expression, regulates microglia activity and KP metabolic balance, produces neurotoxic metabolites and reduces neuroinflammation, and promotes the production of neuroprotective metabolites (Brooks et al., 2017). Bordeleau et al. revealed that desipramine can reduce inflammatory responses, regulate KP, and improve depressive symptoms by reducing the expression of IDO1 and IDO2 in microglia (Bordeleau et al., 2019). KP is involved in the CNS inflammatory response. In the CUMS model, Cang-ai volatile oil reduces the level of pro-inflammatory factors (IL-6, IL-1β, TNF-α, IFN-γ), increases the level of anti-inflammatory factors (IL-4, IL-10), regulates tryptophan metabolic pathways, reduces KYN, QUIN and KYN/Trp ratio, increases KYNA levels, inhibits KP activity, thereby improving OFT, FST and SPT behavioral performance (Zhang et al., 2021).

**FIGURE 6:**
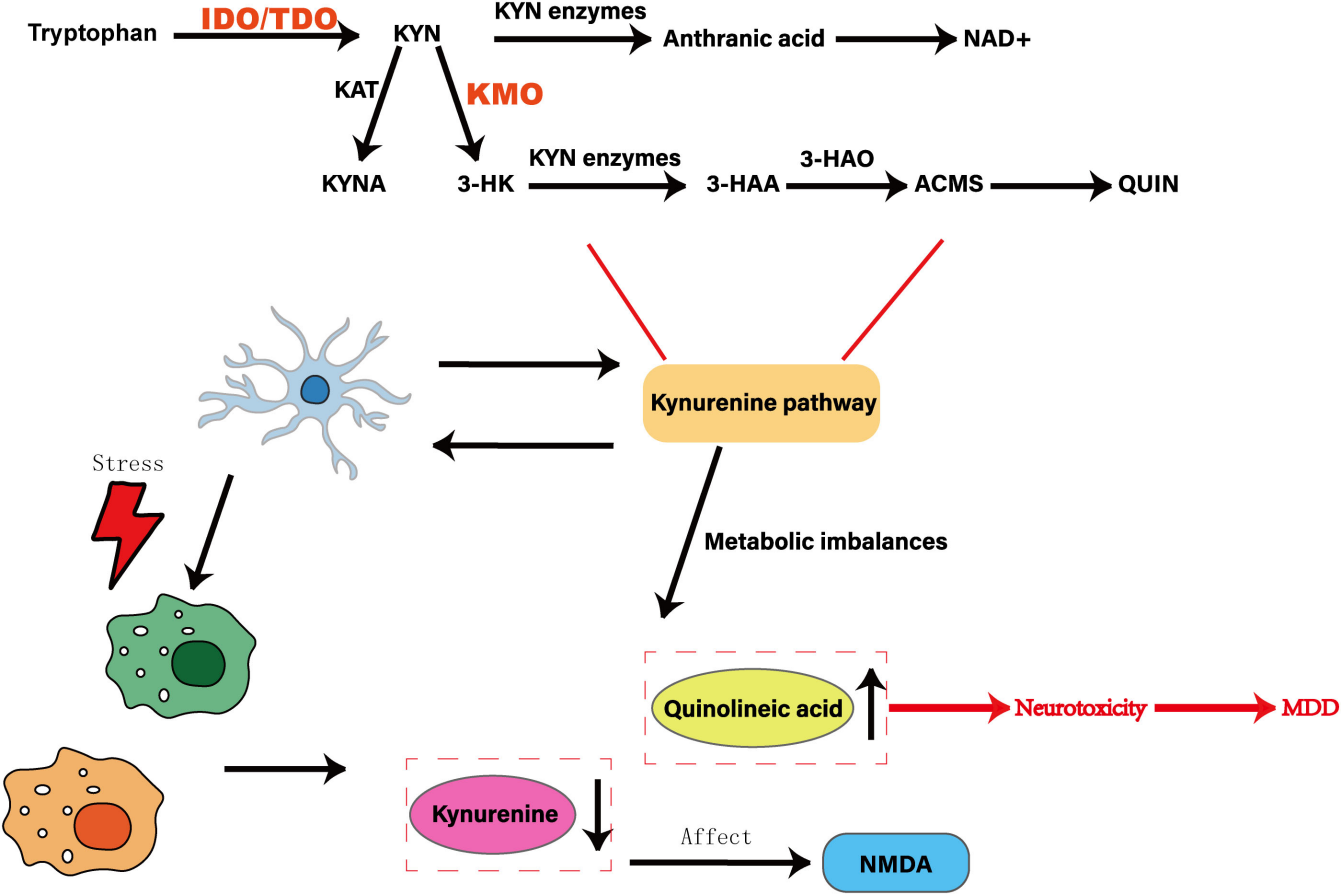
Effect of abnormal activation of microglia on the kynurenine pathway. Tryptophan is shown as a precursor to the KYN pathway, which is converted to KYN by the rate-limiting enzymes IDO and TDO. KYN is catalyzed by KAT enzyme to generate KYNA, which is an endogenous NMDA receptor antagonist with neuroprotective effects. Under the action of KMO enzyme, 3-HK is generated, which is then converted into 3-HAA by KYN enzyme, oxidized to ACMS by 3-HAO, and further metabolized into QUIN, which is an NMDA receptor agonist with neurotoxicity. The abnormal activation of microglia can trigger an imbalance in KP metabolism, resulting in an increase in the neurotoxic product KIN and a decrease in the neuroprotective product KYNA, which in turn produces neurotoxic substances, which are closely related to MDD.

Chronic stress and inflammation cause the upregulation of IDO expression in the brain. The tryptophan metabolite quinolinic acid mediated by it activates the NLRP3 inflammatory body of microglia, causing microglia to abnormally devour synaptic components, damage synaptic plasticity, and promote the development of MDD (Li S. et al., 2022). However, there are many shortcomings in current research. It is unclear whether quinolinic acid produced by IDO in microglia is mediated by NLRP3 inflammatory bodies. The mechanisms of quinolinic acid regulating microglia function and affecting synaptic stability and plasticity in the IDO-KP pathway also need to be further explored. The specific mechanism by which microglia mediates KP metabolic imbalance and affects depressive behavior is unclear. There are limitations in regulating KP metabolic imbalance by activating or inhibiting microglia alone. The functional status of microglia itself has not been clarified, and microglia itself has not been deeply explored., resulting in insufficient research on the microglia-KP axis. Whether other mediators are added between the microglia-KP axis is still unknown. The order of inflammation, microglia activation and KP imbalance in the process of inflammation-mediated MDD is also the focus of future research. It is of great significance to deeply delve into MDD itself and the metabolic products between microglia and KP. As shown in Figure 6. For example, Chen et al. measured CSF quinolinic acid in a cohort of only 20 MDD patients, limiting statistical power and leaving sex differences unexplored (Chen et al., 2021). Norden et al. (2015) reported that a KMO inhibitor reverses depressive-like behavior in rodents but did not confirm central target engagement or rule out peripheral off-target actions. These gaps underscore the need for larger, sex-balanced human cohorts and for PET ligands that directly quantify brain KMO activity *in vivo*. Building on HPA-axis and gut-derived cytokine fluxes, microglial IDO induction emerges as a convergent node where stress hormones and microbial metabolites jointly steer tryptophan metabolism toward either neuroprotection or neurotoxicity. Peripheral kynurenine-to-tryptophan ratios poorly predict brain levels of neurotoxic QUIN or neuroprotective KYNA, in part because of region-specific blood–brain barrier transport and variable enzyme expression. Most KMO inhibitor studies report efficacy in young rodents but fail to assess brain penetrance or off-target effects in primates. Genetic association studies linking IDO polymorphisms to MDD risk are underpowered (*n* < 500) and lack replication. To overcome these limitations, future research should pair CSF and plasma metabolomics in large, well-phenotyped cohorts and develop PET ligands for KMO and IDO to enable *in vivo* mapping of pathway flux. Rodent stress models consistently report elevated microglial IDO activity, increased quinolinic acid (QUIN) accumulation and corresponding behavioral deficits. In contrast, human CSF and plasma studies yield more variable results: while some cohorts show higher QUIN/KYNA ratios in MDD patients, others find no significant difference or even reduced QUIN levels after antidepressant treatment. These discrepancies may stem from species-specific IDO regulation, differences in blood–brain barrier permeability, and clinical heterogeneity. Recognizing these conflicts underscores the need for paired CSF–plasma metabolomics in large, well-characterized human samples to validate mechanisms first identified in animals.

Collectively, chronic stress–induced HPA-axis activation, gut-derived microbial signals and pro-inflammatory cytokines converge on microglial receptors—GRs, β-adrenergic receptors, TLRs and IDO—to recalibrate microglial phenotypes from homeostatic to pathogenic. Primed microglia then perpetuate neuroinflammation via IL-1β/IL-6 release, drive kynurenine flux toward neurotoxic quinolinic acid, and compromise synaptic and white-matter integrity. Simultaneously, microglia-mediated disruption of the gut-barrier and increased peripheral immune tone feed back to the CNS, closing a multisystem loop. This nexus positions microglia as the central integrator of neuroendocrine, immune, metabolic and microbiome inputs in MDD.

## 4 Modifiers

### 4.1 Sex difference

Global epidemiological data on MDD show a higher incidence among women (5.1%) than men (3.6%), with women accounting for 65% of the total number of MDD patients. However, studies suggest that men and women The actual incidence of MDD may be similar, with women reporting mild to moderate symptoms and men reporting severe symptoms and suicidal tendencies (Shi et al., 2021). This may be related to the fact that women are more susceptible to estrogen fluctuations and social pressure and are more likely to confide, while men are more likely to deny depressive symptoms, accumulate negative emotions, and seek help only when severe. From a neurobiological perspective, female patients with MDD have impaired LTD-like plasticity in the left motor cortex, although their peripheral BDNF concentrations are comparable to healthy subjects. This plasticity defect may be closely related to the pathophysiology and treatment mechanism of the disease (Yu et al., 2020).

Microglia differ in shape and function among individuals of different sexes (Nelson et al., 2019), which may be one of the reasons why women are more susceptible to MDD (Liu L. et al., 2019). Carrier et al. found that in women, the association between microglia function and BMI, financial insecurity, and the association between prenatal MDD symptoms of the mother and offspring was significantly modulated, while in men the association was not significant (Carrier et al., 2024). Liu L. et al. (2019) studied male and female mice through CUMS and found that female mice were more sensitive to stress and showed more pronounced depression-like behavior. In addition, the expression of pro-and anti-inflammatory factors in the hippocampus was gender-specific, and the expression of BDNF and its receptor TrkB decreased more in female mice (Bollinger et al., 2017). Pohl et al. found in 2017 that there are differences in the activation and morphology of microglia between male and female rats, and stress can gender-specifically change their density, morphology and immune factor expression (Pohl et al., 2021). In 2019, they further discovered that gonadal hormones such as estrogen and testosterone can affect the development of MDD by regulating the stress response of microglia in the medial prefrontal cortex (Maitra et al., 2023). In addition, Yang et al. found that in prairie voles, there were gender differences in the response of microglia to a stressful life event such as losing a partner (Yang et al., 2024). In the CUMS-induced rat model of MDD, female rats showed more pronounced depression-like behaviors such as reduced sugar water preference and increased immobility time, with significant increases in their GCs levels and pro-inflammatory microglia markers, and decreased levels of 5-HT and NE in the hippocampus (Fitzgerald et al., 2024). A single RNA sequencing of the dorsolateral prefrontal cortex of more than 160,000 nuclei from 71 female and male donors showed that the expression pattern of MDD-related genes was generally similar between the sexes, but there were gender differences in significantly differently expressed genes. Microglia and parvalbumin interneurons contributed most in women, while deep excitatory neurons, astrocytes and oligodendrocyte precursors contributed most in men (Xia et al., 2022). This suggests that microglia may combine with other glia cells to promote gender susceptibility to MDD. However, sequencing results alone are not enough to fully reveal the role of microglia and other cell types in gender differences in MDD, and further verification is still needed in conjunction with more experimental means and research methods.

### 4.2 Genetic and environmental factors

MDD is a polygenic inherited disease that is closely related to polymorphisms in genes such as SLC6A4, BDNF, ANK3 and CACNA1C. The SLC6A4 gene encodes the 5-HT transporter, and its short allelic variation will reduce the expression of the 5-HT transporter and affect emotional regulation (Pezawas et al., 2008). The BDNF gene encodes brain-derived neurotrophic factor. Its Val66Met polymorphism can reduce the intracellular transport efficiency of BDNF protein, reduce BDNF levels in key brain areas such as the hippocampus, and weaken neurogenesis and synaptic plasticity. However, in gene-environment interaction studies, the Met allele of the BDNFVal66Met polymorphism can alleviate the relationship between life stress and depression (Colle et al., 2017). ANK3 gene encodes an anchor protein that affects neuronal function. Its rs10994359 polymorphism protects against cortical thickness reduction in healthy individuals, but this protection is lost in first-episode MDD patients, suggesting that this gene may affect cortical aging in MDD (Cheng et al., 2020). The CACNA1C gene encodes the α1 subunit of the calcium channel. Its mutation can increase the concentration of intracellular calcium ions, leading to excessive excitation of neurons and imbalance of the neurotransmitter system. Moreover, the rs1006737 polymorphism of this gene has significant gene-environment interactions with threatening life events in MDD, increasing the risk of MDD (Zhao et al., 2019). At the same time, reduced levels of Cacna1c weaken the function of the midbrain limbic dopamine system (Terrillion et al., 2017). Environmental stress can lead to over-activation of the HPA axis by inducing DNA methylation of the NR3C1 gene. The methylation level in the promoter region of this gene in patients with MDD is 20%–30% higher than that in healthy people. MDD is clustered in families, with the incidence of one parent having the disease rising to about 25%, and the incidence rising to 50%–75% in cases where both parents have the disease. Individuals have different genetic susceptibility, and those carrying multiple genetic variants are at higher risk. Living environment, psychological quality, etc. interact with genetic factors to affect the disease. A systematic review and meta-analysis of the risk of MDD in fathers and descendants found that father depression increases the risk of depression in children by 42% (Dachew et al., 2023). These genetic variants act together with environmental factors to affect an individual’s susceptibility to MDD by affecting the neurotransmitter system and neuroplasticity.

Genetic and environmental factors may increase the risk of MDD by affecting the function of microglia (Baek et al., 2023). Genetic factors determine the gene expression pattern and functional state of microglia. Certain genetic mutations may make microglia more easily activated and cause neuroinflammation. Environmental factors such as stress, infection, and trauma in early life can also affect the function of microglia, causing them to release pro-inflammatory cytokines, disrupt the neurotransmitter system and neural plasticity, and thus trigger depression-like behavior. Research by GosselinD and his team revealed that the gene expression and functional status of microglia are significantly affected by environmental factors and epigenetic regulation. They respond to environmental changes through changes in environment-dependent transcription networks and epigenetic landscapes, which in turn affect the occurrence and development of neuropsychiatric diseases, including MDD (Zhao et al., 2023). In a mouse model of depression, baicalein reduces hippocampal microglia activation by inhibiting the NF-κB pathway, reduces pro-inflammatory cytokine expression, increases SLC6A4/SERT concentrations, and improves glutamate metabolism, thereby reducing depression-like behavior in open-field tests, elevated cross maze, forced swimming test, and tail suspension test (Du et al., 2019). BDNF is the target of DNA methylation, and its gene expression is regulated by DNA hydroxymethylation (the conversion of 5-mC to 5-hmC through Tet enzyme). In the LPS-induced depression model, the expression of Iba-1 in the hippocampus is increased, the levels of Tet1/2/3 and 5-hmC are down-regulated, and the expression of BDNF mRNA is reduced. These changes induce depression-like behaviors such as behavioral despair and anhedonia in mice, suggesting that depression-like behavior induced by neuroinflammation is closely related to the decrease in hydroxymethylation level of the BDNF gene in the hippocampus (Alboni et al., 2016). Alboni S and others found that in chronic stress models, the impact of fluoxetine on inflammatory response and microglia function is regulated by the living environment, pro-inflammatory in the enrichment environment and anti-inflammatory in the stress environment (Gu et al., 2021). In the CUS model, enrichment environment improves depression-like behavior in SPT, FST, and OFT by regulating rat hippocampal microglia phenotype (decreasing the M1-type marker iNOS and increasing the M2-type marker CD206), inhibiting NF-κB activation and upregulation of pro-inflammatory cytokines (Li Y. et al., 2022). Other studies have found that genetic and environmental factors of MDD are mediated through the kynurenine pathway of tryptophan metabolism. High promoter alleles of pro-inflammatory cytokine genes (IFNG and TNF-α) can lead to the up-regulation of IDO in microglia, which in turn determines genetic susceptibility, and environmental stress affects this pathway by activating TDO (Oxenkrug, 2010).

Although regulating the epigenetic status of microglia and optimizing the living environment can effectively reduce depressive symptoms, current research focuses on a single time point or specific stage, and lacks systematic research on the dynamic changes of microglia throughout the disease process. The specific mechanism of microglia in the occurrence, development and outcome of depression remains unclear. Current research focuses on the impact of single factors (such as specific genetic mutations or single environmental stress) on microglia function, while ignoring the combined role of genetic and environmental factors. The pathogenesis of depression is complex, and it is difficult to fully explain its pathological process based on single factor studies. Although the importance of gene-environment interactions has been confirmed, the specific molecular and cellular mechanisms remain unclear. Future research needs to deeply explore the dynamic changes of microglia throughout depression and their interactions with genetic and environmental factors, reveal their specific mechanisms in the pathological process, and provide a basis for developing more effective treatment strategies.

### 4.3 Microbiota-gut-brain axis

While chronic stress and glucocorticoid-primed microglia drive systemic inflammation, emerging evidence shows that gut microbial metabolites feedback onto microglia—modulating their activation state and linking peripheral dysbiosis to central neuroimmune alterations. The microbiota-gut-brain axis is a complex two-way communication system covering the central nervous system, intestinal flora, neurotransmitters, immune regulation, endocrine and metabolic products (Kuwahara et al., 2020). Under physiological conditions, neurotransmitters (such as Glu, GABA, NE, DA, 5-HT) and metabolites (such as BA, SCFA, Trp) synthesized by intestinal flora affect intestinal cell functions and the synthesis and secretion of neurotransmitters, thereby regulating intestinal motor function and brain behavior (Dicks, 2022). The vagus nerve (VN) serves as the main neural pathway that integrates intestinal signals to the brain and feeds brain signals back to intestinal epithelial cells. Its signal transmission relies on intestinal nervous system (ENS) neurons rather than directly on the intestinal wall or intestinal microbiota (Dicks, 2023). The hypothalamus communicates with intestinal epithelial cells through the HPA axis, and the regulation, development and renewal of ENS neurons are controlled by the intestinal microbiome (Farzi et al., 2018). Th17 cells and Treg cells play an important role in maintaining intestinal and extraintestinal immune homeostasis. The balance of Th17 cells and Treg cells is regulated by intestinal flora, and may affect brain immune activity and maintain homeostasis (Wang et al., 2024).

Microbiome-gut-brain axis homeostasis disorders associated with MDD onset (Chen et al., 2024). Intestinal flora imbalance can inhibit SCFA synthesis, destroy intestinal barrier, make inflammatory factors enter blood circulation and break through blood-brain barrier, activate neuroinflammation, interfere with neurotransmitter metabolism, disrupt immune network, lead to depression and cognitive impairment in patients, and aggravate depressive behavior (Zhao H. et al., 2022). Studies have found that changes in the intestinal flora of patients with MDD (increases in Deinococcaceae, Deinococci spp., and Odoribacter spp., and decreases in Bacteroidaceae, Turicibacteraceae, Clostridiaceae, Pasteurella spp., Bacteroides spp., Alistipes spp., Turicibacter spp., Clostridia spp., Rothia spp. and Enterobacter spp.) are associated with inflammatory factors (such as hsCRP, IL-6) and cognitive function indicators (such as CCT1, delayed memory) (Liu P. et al., 2021). A randomized clinical trial found that probiotics act as adjunctive treatment for patients with MDD by improving the microbiota-gut-brain axis. The results showed that at weeks 4 and 8, the HAMD-17 score and HAMA score decreased significantly. After linear mixed model analysis, the normalized effect size showed that probiotics were significantly effective in improving depressive symptoms and anxiety symptoms (Nikolova et al., 2023). In the CUMS model, matrine regulates the microbiota-gut-brain axis, improves the disorder of intestinal flora and metabolites, restores intestinal permeability, reduces intestinal inflammation, reduces the level of pro-inflammatory cytokines, and increases the level of BDNF in the brain, thereby alleviating depression-like behavior in mice, which is manifested by increasing SPT sugar water consumption, reducing FST and OFT immobility time, and shortening the latency period of NSFT (Zhang et al., 2023).

Studies have shown that the interaction between the microbiota-gut-brain axis and microglia is an important link in the pathogenesis of neuroinflammation and neurodegeneration (Loh et al., 2024). Regulating the intestinal microbiota can improve the activation of microglia, which in turn delays the progression of MDD. In the CUMS model, rifaximin regulates intestinal microbiota and SCFA levels, increases the relative abundance of Ruminococcaceae and Lachnospiraceae, increases butyrate content in the brain, reduces the pro-inflammatory function of microglia, and promotes the release of anti-inflammatory factors, improving sucrose preference in adolescent rats, open-field testing, and depression-like behavior in the Morris Water Maze (Tian et al., 2019). Environmental pollutants may also affect microglia through the microbiota-gut-brain axis. For example, polybrominated diphenyl ethers (BDE47) are a common environmental pollutant that can enter the body through the food chain and cross the blood-brain barrier. Research by Schmidtner et al. has shown that BDE47 can disrupt the balance of intestinal flora, increase the release of inflammatory factors, activate microglia, and then affect the DA system, leading to neuroinflammation and cognitive dysfunction (Schmidtner et al., 2019). In recent years, the potential role of antibacterial drugs in the treatment of MDD has attracted increasing attention. Schmidtner et al. (2019) found a decrease in the number of microglia cells in the prefrontal cortex in a rat model of high anxiety behavior, accompanied by anxiety and depression-like behavior. Further research has shown that minocycline treatment can increase the abundance of butyric acid-producing bacteria, increase plasma 3-OH-butyric acid levels, and reduce depressive symptoms by reducing microglia density and regulating the intestinal microbiome (Huang et al., 2023). This suggests that antibacterial drugs may improve depression-related behaviors by regulating intestinal microbiota and microglia function. In addition, studies have also found that loss of the intestinal microbiota changes the transcriptome of microglia cells, which in turn affects depression-like behavior. These changes can be reversed through microbiota colonization, regulating the transformation of microglia subsets, and improving behaviors related to depression and cognitive dysfunction (Brown et al., 2022).

At present, although research on treating MDD by regulating the microbiota-gut-brain axis to affect the function of microglia has shown potential application value, most of them are limited to animal experiments, and data on microglia in clinical research is scarce. Animal experimental studies also have limitations such as insufficient long-term efficacy evaluation and unclear impact of individual differences. With the continuous development of visualization technology, in-depth exploration of the interaction mechanism of the human microbiome-gut-brain axis will lay a solid theoretical foundation for clinical translational applications in this field. Microbiome profiling studies in MDD are confounded by diet, antibiotics and sampling methods, which can drastically alter SCFA measurements and microbial composition. The heterogeneity of 16S rRNA pipelines also impedes cross-study comparisons. Fecal transplant experiments demonstrate causality in rodents but are difficult to standardize and ethically complex in humans. Standardized feeding protocols, shotgun metagenomic sequencing and the integration of metabolomic readouts are therefore necessary to delineate how specific microbial metabolites regulate microglial phenotypes.

## 5 Treatment significance and future strategies

### 5.1 Inhibiting M1 microglia activation for treatment of MDD

Studies have shown that in the IFN-α-induced MDD model, microglial activation is closely related to depression-like behaviors such as FST, TST, and new object recognition tests, manifested by increased expression of pro-inflammatory markers of M1 polarization (such as MHC-II, CD86, CD54). These changes are particularly significant in susceptible mice (Wang Y. et al., 2018). In a CMS-induced depression model, microglia activation is accompanied by HIP neuroinflammation and depression-like behavior (Guo et al., 2023). Therefore, inhibiting MI-type microglia activation becomes a potential strategy for the treatment of MDD. In the LPS depression model, insulin-like growth factor 2 (IGF2) reduces neuroinflammation, protects HIP neurogenesis, increases sucrose preference in mice, increases exploratory activity and reduces immobility time by regulating microglia polarization state (inhibiting M1 type activation and promoting M2 type transformation) and the TLR4/MyD88/NF-κB signaling pathway (Peng et al., 2021). Feng et al. found that in an LPS-induced depression model, microglia-specific knockout of Dlg1 significantly reduced the number of HIP microglia and the expression of pro-inflammatory factors (iNOS, TNF-α, IL-1β, IL-6) by inhibiting NF-κB and MAPK signaling pathways, and effectively improved depression-like behavior (increasing OFT center activity/total distance, EPM open arm exploration, and reducing TST/FST immobility time) (Feng et al., 2020). In the CMS depression model, mefenamic acid reduces iNOS levels, increases sucrose preference and reduces forced swimming immobility time by inhibiting microglial activation in the hippocampal DG region (reducing Iba1/CD68 expression) and ERK1/2 and P38MAPK signaling pathways (Liu C. et al., 2021). miR-146a reduces TST and FST immobility time and increases sucrose preference by inhibiting microglia activation and inflammatory factor (Iba-1, iNO, IL-1β, TNF-α, IRAK1, TRAF6, NF-κ Bp65) expression in LPS and CUMS models (Yu et al., 2023). Bian et al. found that low-dose LPS pretreatment effectively improved the total travel distance and speed in OFT and increased the TST and FST immobility time by activating GPR84-dependent immune tolerance mechanism, inhibiting the activation of M1 microglia and the release of proinflammatory factors (TNF-α, IL-1β, IL-6) in HIP region (Bian et al., 2021). In LPS-induced depression model, RGFP966 reduces microglial activation and neuroinflammation by inhibiting HDAC3/TLR4/NLRP3 signaling pathway, and improves depression-like behaviors such as OFT and FST (Zhou et al., 2024). Cynaroside inhibits M1 polarization of microglia, reduces lipid peroxidation and iron death by regulating IRF1/SLC7A11/GPX4 signaling pathway in LPS model, and exhibits antidepressant effect similar to fluoxetine in CUMS model, effectively improving SPT, OFT, TST, FST, EPM, MBT and other depression-like behaviors (Wang et al., 2023). Wang et al. (2023) found that Saikosaponins B2 inhibits microglia activation by regulating the TLR4/NF-κB signaling pathway, reduces LPS-induced neuroinflammation, improves CUMS-induced depression-like behavior, and at the same time reduces iron death in a GPX4-dependent manner. Maintain calcium homeostasis, relieve endoplasmic reticulum stress, and further inhibit microglia activation (Valenza et al., 2024). These findings suggest that inhibiting M1 microglia activation and its associated inflammatory response is a potentially effective strategy for the treatment of MDD.

### 5.2 Promotion of M2 microglia activation for MDD

In the early stage of MDD, moderate activation of microglia may be an adaptive response to stress stimuli, contributing to the maintenance of internal homeostasis (Zhu et al., 2024). However, chronic uncontrolled stress may lead to impaired microglial function, exacerbating the development of MDD. In this case, pharmacological intervention to restore normal physiological functions of microglia, such as promoting their proliferation and activation, may be beneficial for the treatment of MDD (Afridi and Suk, 2023). Recent studies suggest that the role of microglia in MDD is not limited to its role in neuroinflammation, but also includes their function in regulating neurogenesis, synaptic plasticity, and neural network formation, all of which are key factors in the pathophysiology of MDD. By regulating the activation state of microglia, neuroinflammation can be reduced and neuroprotection promoted, which may improve MDD symptoms (Böttcher et al., 2020). In addition, microglia in MDD patients have been found to exhibit a non-inflammatory phenotype by single-cell mass spectrometry and are associated with increased expression of homeostatic markers, which may reflect a protective role of microglia in MDD (Ji et al., 2022). Pharmacological activation of microglia has been shown to alleviate depression-like symptoms in animal models. Studies have shown that stimulation of microglia proliferation by endotoxin, macrophage colony-stimulating factor, or granulocyte-macrophage colony-stimulating factor partially or completely reverses depression-like behavior in mice after 5 weeks of CUS exposure (Brites and Fernandes, 2015). Another study explored the role of microglia in the pathogenesis of MDD, including their activation in stress responses and how they regulate pathways associated with depression by secreting extracellular vesicles (EVs) and microRNAs (miRNAs) (Wu G. et al., 2023). Banxia-houpo Decoction significantly promoted microglia polarization to M2 (up-regulating Arg1/Ym1/Fizzl1 expression and increasing IL-4/IL-10 secretion), inhibited M1 activation (decreasing IL-6/TNF-α/IL-1β levels), decreased Iba1 + cell number and improved cell morphology by activating TrkA/Akt/CREB/Jmjd3 signaling pathway, thereby effectively alleviating depression-like behaviors (improving OFT, SPT, NSFT, TST and FST performance) in CUMS depression model (Liu et al., 2023).

### 5.3 Transformation outlook

#### 5.3.1 Antidepressants approved for clinical use

Antidepressants play a role in the treatment of MDD by regulating microglia activity through a variety of mechanisms, including anti-inflammatory effects, affecting neuroplasticity and regulating neurotransmitter systems. Currently, antidepressants approved for clinical use include selective 5-HT reuptake inhibitors (SSRIs), 5-HT and NE reuptake inhibitors (SNRIs), monoamine oxidase inhibitors (MAOIs), tricyclic antidepressants (TCAs), DA receptor agonists, etc.

SSRIs include fluoxetine, paroxetine, sertraline, citalopram, etc., which alleviate depressive symptoms by inhibiting presynaptic membrane 5-HT operating protein, preventing 5-HT reuptake, increasing 5-HT concentration in synaptic gap, activating postsynaptic membrane receptors, enhancing neural signal transmission, regulating neural plasticity, improving emotional and cognitive functions, etc (Gałecki et al., 2018). However, SSRIs have recently been found to modulate many targets outside the 5-HT system. In a mouse model of LPS-induced depression, fluoxetine reduced neuroinflammation by inhibiting microglial activity, decreased IL-1β, TNF-α levels in HIP, decreased Iba-1 expression, increased NA, BDNF, and dendritic spine density, and improved depression-like behavior in OFT, EPM, TST, and FST (Hong et al., 2024). The antidepressant effect of paroxetine depends on mTOR signaling in HIP in the CUMS and CSDS models (Xu D. et al., 2018). It protects astrocyte L-Glu transporter from early downregulation in neuroinflammation by inhibiting L-Glu release from activated microglia in LPS model (Fujimori et al., 2015). Sertraline exerts anti-inflammatory and antidepressant-like activities by inhibiting TNF-α, IL-1β-induced NF-κB activation, blocking TNF-α/TNFR1 signaling, and reducing pNF-κ B p65, pIκB-α and downstream inflammatory factors iNOS, TNF-α, IL-1β levels in central tissues and microglia in CUMS model (Lu et al., 2019). In LPS+INFγ model, fluoxetine and S-citalopram inhibited the expression of CD86, decreased TNFα, IL-1β, IL-6 and iNOS mRNA levels in primary microglia, and increased the expression of CD206, IL-10mRNA in IL-4 model, thus regulating immune system (Su et al., 2015).

SNRIs include venlafaxine, duloxetine, etc., which inhibit 5-HT and NE reuptake, increase their concentration, enhance neural signal transmission, regulate related neural circuit function, and improve symptoms such as depression in MDD patients (Li et al., 2020). In CUMS+ sleep deprivation model, venlafaxine combined with melatonin increased sugar consumption rate, learning, memory and exploration desire of depressed rats by up-regulating caspase-3 expression in pineal glial cells and hippocampal microglial cells, down-regulating iba-1 expression, reducing CREB, GDNF and Spinoline secretion, improving dendritic and spiny damage in hippocampal DG region (Li Z. et al., 2022). Duloxetine inhibited the expression of NO, iNOS, P-IκBα, P-Akt and Akt proteins in BV-2 microglia cells in LPS model and exerted anti-inflammatory effects (Nakatani et al., 2022).

MAOIs, including tranylcypromine and phenelzine, exert antidepressant effects by inhibiting the activities of MAO-A and MAO-B and preventing the degradation of 5-HT, NE and DA. The results showed that long-term administration of tranylcypromine and 4-fluorotranylcypromine significantly affected the levels of monoamine neurotransmitters and their metabolites in the brain of rats, increasing the contents of NE, 5-HT and DA in the frontal cortex, nucleus Accumbens, caudate nucleus and HIP, and decreasing the contents of their acid metabolites 5-HIAA, DOPAC and HVA, suggesting that the drugs might inhibit the activity of monoamine oxidase to reduce the degradation of monoamine neurotransmitters and make them accumulate in the brain (Greenshaw et al., 1989). Phenelzine is effective against refractory and senile MDD, but increases inflammation in an LPS-activated microglial model, suggesting that its antidepressant effects may be mediated through non-microglial pathways (Chung et al., 2012).

TCAs, including amitriptyline, doxepin, imipramine, etc., exert antidepressant effects by inhibiting NE and 5-HT reuptake. Amitriptyline induces the expression of various BDNF mRNA variants in primary cultured rat cortical astrocytes and microglia through MEK/ERK signaling pathway, up-regulates the expression of exon I, IV and VI, and increases the level of BDNF mRNA (Hisaoka-Nakashima et al., 2016). Amitriptyline and doxepin significantly reduced inflammation, inhibited microglial activation, but did not affect TNF-α and IL-6 levels, and were non-glial cytotoxic in an IFN-β pretreated astrocyte-microglial co-culture model, indicating that they did not interfere with the anti-inflammatory effects of IFN-β and had compatible benefits (Faustmann et al., 2023). In a model of repeated social frustration, imipramine blocks bone marrow mononuclear cell migration to the brain by inhibiting the HPA axis (lowering corticosterone and IL-6) (Ramirez and Sheridan, 2016), blocking bone marrow monocyte migration to the brain, and downregulating microglia activation (decreasing IL-6/TNF-α/IL-1β expression), demonstrating its antidepressant effect through neuroimmunomodulation (Ramirez et al., 2015).

DA receptor agonists include palmatine, pramipexole, ropinirole, bromocriptine, etc. Palmatine, also known as memantine, acts as an NMDA receptor antagonist and exerts synergistic antidepressant effects by indirectly regulating DA system function and inhibiting neuroinflammation. Palmatine significantly inhibited activation of hippocampal M1 microglia (down-regulation of TNF-α, IL-6, CD68 and iNOS) and promoted M2 polarization (up-regulation of IL-4, IL-10, CD206, Arg1 and Ym1) in LPS-induced depression model by modulating PDE4B/KLF4 signaling pathway, manifested by increased sucrose preference rate, shortened immobility time in forced swimming and tail suspension test, thus effectively improving depression-like behavior (Wang L. et al., 2022). Recent studies have shown that pramipexole, ropinirole, bromocriptine and other direct DA receptor agonists improve core symptoms of depression by activating the midbrain limbic reward pathway and prefrontal cortex D2/D3 receptors, but their direct effects on microglial polarization have not been reported (Zhao F. et al., 2022).

Furthermore, in LPS models, the tetracycline antibiotic minocycline inhibits microglial M1 polarization (decreases iNOS/COX-2/IL-1β) and promotes M2 polarization (enhances anti-inflammatory and phagocytic functions) by activating SIRT1 signaling pathway, while down-regulating acetylated p53 and STAT1 inflammatory signals, thereby reducing neuroinflammation and exerting antidepressant effects (Verdonk et al., 2019). Ketamine, as an NMDA receptor antagonist, promotes synaptic plasticity by blocking NMDA receptors, activating AMPA-mTOR-BDNF pathway, and regulating microglial polarization to achieve rapid antidepressant effects. In the CUMS depression model, ketamine reduces the neuroinflammatory response by specifically regulating the polarization state of microglia in the HIPCA3/DG region (inhibiting M1-type activation: reducing the expression of TNF-α, IL-1β and IL-6; promoting M2-type polarization: increasing IL-10 expression), thereby improving anhedonia, decreased exercise activity and behavioral despair in mice (Edem et al., 2022). In the LPS depression model, ketamine inhibits microglia polarization through the HMGB1-RAGE axis (inhibits M1-type pro-inflammatory polarization and promotes M2-type anti-inflammatory polarization), increases autophagy flux, and improves depression-like behavior (increased sucrose preference, reduced FST, TST, and NSFT immobility times) (Wu M. et al., 2022).

#### 5.3.2 Drugs undergoing preclinical trials

Antidepressant drug candidates currently undergoing preclinical research mainly focus on new mechanisms of action, including TSPO ligands and PDE4 inhibitors that target microglial polarization, brain-targeted monoclonal antibodies that block pro-inflammatory factors such as IL-6/IL-1β, mGluR2/3 negative allosteric regulators and AMPA receptor agonists that regulate the glutamatergic system, TrkB small molecule agonist 7,8-DHF derivatives that mimic BDNF function, HDAC6 selective inhibitors that specifically regulate epigenetics. as well as specific probiotic strains that act through the microbial-gut-brain axis (such as Lactobacillus reuteri NK33). In LPS model, TSPO ligand XBD173 exerts neuroprotective effects by inhibiting the expression of LPS-activated microglia proinflammatory factors (CCL2/IL6/iNOS) and reducing neurotoxicity (Karlstetter et al., 2014). TSPO ligand ZBD-2 (XBD173 derivative) improves depression-like behavior in a mouse model of postpartum MDD by up-regulating TSPO expression in the basolateral amygdala, regulating HPA axis function, enhancing 5-HT and BDNF secretion, and maintaining synaptic protein balance (Li et al., 2018). In the LPS model, the new PDE4 inhibitor FCRP03 significantly reduced the release of pro-inflammatory factors (TNF-α/IL-1β/IL-6) from microglial cells in the HIP and cortex of mice by activating the cAMP/PKA/CREB signaling pathway and inhibiting NF-κB activation, showing a strong anti-neuroinflammatory effect (Zou et al., 2017). In the CUMS model, PDE4 inhibitors effectively reverse model-induced microglia activation and morphological abnormalities, and reduce IL-1β release by inhibiting the HMGB1/RAGE/TLRP4 signaling pathway and NLRP3 inflammasome activation (reducing the expression of HMGB1, RAGE, TLRP4, p-p38MAPK, NF-κB and NLRP3/ASC/Caspase-1), thereby exerting an antidepressant effect (Xie et al., 2021). IL-6 is a potential diagnostic marker of MDD. In an LPS-attacking depressive model, its receptor antagonist Interleukin-6 effectively improves depressive behavior and reverses overactivation of glial cells by inhibiting peripheral/central inflammation (reducing IL-6/CRP/TNF-α), regulating HPA axis activity (reducing CRH/CORT), and repairing HIP synaptic plasticity (activating the Wnt/β-catenin pathway) (Xu et al., 2023). In a rat model of diabetic depression, the mGluR2/3 antagonist LY341495 inhibits the Glu-mGluR2/3-ERK signaling pathway, improves HIP neuron apoptosis (reduction of TUNEL positive cells and reduction of caspase-3 expression), reduces immobility time in FST, alleviates spatial memory deficits, and increases exploration activity (Liu Z. et al., 2019). In an elderly rat model, the AMPA receptor positive allosteric modulator S47445 shows the utic potential to improve cognitive function and antidepressant-like effects by upregulating the expression of BDNF, NT-3 and NGF in the prefrontal cortex and HIP (Calabrese et al., 2017). Stagni et al. (2017) found that the TrkB receptor agonist 7,8-DHF can significantly increase the number of neural precursor cells and granulosa cells by promoting HIP neurogenesis, and at the same time enhance synaptic plasticity, as reflected in the increase in dendritic spine density and synaptophysin expression levels, improving HIP-dependent learning and memory functions. Jochems and other researchers have developed new highly selective HDAC6 inhibitors ACY-738 and ACY-775. These compounds have excellent brain bioavailability and can significantly enhance the acetylation of α-tubulin in the brain without affecting histone acetylation. It has shown an antidepressant effect in tail suspension tests and social frustration models, and this effect relies on central HDAC6 inhibition. It can also enhance the antidepressant activity of the SSRI drug citalopram (Jochems et al., 2014). Han et al. (2020) found that Lactobacillus reuteri NK33 and Bifidobacterium elongatus NK98 effectively alleviated the depressive behavior of mice induced by Escherichia coli K1 by regulating intestinal flora (reducing Proteobacteria/increasing Clostridia), increasing hippocampal BDNF/NeuN expression, and inhibiting NF-κB activation. These drugs have shown the potential to improve depression-like behavior in animal models, but about 70% of drug candidates fail in the clinical transformation stage due to problems such as insufficient blood-brain barrier penetration or toxic side effects. The current research and development strategy is shifting to the development of multi-target drugs that have anti-inflammatory, neurotrophic and neuroplasticity regulatory effects, in order to break through the limitations of existing antidepressants.

#### 5.3.3 Identification of potential biomarkers

Biomarker research for MDD has formed a multi-dimensional system including peripheral biochemistry, neuroimaging, microbiome and genetic markers. Peripheral biochemical indicators (CRP, serum CC chemokines, plasma nesfatin-1 and cortisol levels, KYNA/QUIN ratio) showed abnormalities in inflammation, HPA axis, and KP metabolic pathways; neuroimaging characteristics were abnormal in TSPO-PET imaging (enhanced [11C] PK1195, hippocampal volume, default mode network connectivity) reflecting changes in brain function and structure; microbiome and genetic markers (BDNFrs6265, miR-132) suggested that abnormalities in microbiota-gut-brain axis and epigenetic regulation also have diagnostic potential.

Studies have found that peripheral inflammation levels (CRP > 3 mg/L) in patients with MDD are significantly correlated with reduced striatum reward response, suggesting that inflammation may participate in the pathogenesis of depression by affecting the reward system (Burrows et al., 2021). The serum CC chemokine spectrum of MDD patients was abnormal (MIP-1α/β increased, MCP-1/4, TARC, MDC, Eotaxin-3 decreased), among which MIP-1α, MCP-4, TARC, Eotaxin-3 had diagnostic potential (AUC > 0.7), which provided new evidence for the immune dysregulation mechanism of MDD (Gao et al., 2022). The combined detection of plasma nesfatin-1 and cortisol levels could effectively differentiate moderate and severe MDD (AUC = 0.993), with a diagnostic sensitivity of 97.1% and specificity of 98.6%, suggesting that they could be used as a new biomarker combination for MDD (Xu Y. et al., 2018). Büttner et al. (2015) found that markers associated with MCs receptor function (increased aldosterone/cortisol ratio, decreased systolic blood pressure) predict treatment response in MDD patients, with men being particularly sensitive to markers of central MCs receptor activation (decreased slow-wave sleep, increased heart rate variability). Liu et al. (2018) found that plasma KYNA level can be used as a diagnostic marker for MDD (AUC = 82.5%, cut-off value 15.48 ng/ml). When combined with QUIN, the diagnostic accuracy increased to 83.6%, supporting the key role of KP metabolic imbalance in the pathogenesis of MDD (Liu et al., 2018).

Suzuki et al. (2013) found that the binding potential of brain [11C](R)-PK11195 can be used as a representative indicator of microglial activation. [11C] PK11195 PET study showed elevated TSPO nodes in anterior/posterior cingulate gyrus and significantly enhanced microglial activation in HIP region in untreated MDD patients (Joo et al., 2021). HIP structural abnormalities were found to be symptomatic and prognostic in MDD patients, with significantly reduced volumes of the left hippocampal head (CA1, GC-ML-DG, and ML subregions) in patients with anhedonia. The baseline volume of HIP (Ht) decreased in all MDD patients, and larger Ht volume predicted remission after 8/16 weeks of treatment, suggesting that different HIP subregions were associated with core symptoms and treatment response, respectively (Wu C. et al., 2023). Dynamic fMRI analysis based on emotion-face matching task showed that the number of coherent clusters among functional brain networks could effectively predict the change of depression severity at 3 months (accuracy rate 87.5%) and 6 months (77.4%) in MDD patients, among which the dynamic connection pattern of post-default pattern network and dorsal attention network had the most predictive value, suggesting that the dynamic characteristics of brain networks can be used as novel biomarkers for MDD prognosis (Pilmeyer et al., 2024).

Five gut microbiota derived inflammation-related serum metabolites [LysoPC(16:0), deoxycholic acid, docosahexaenoic acid, taurocholic acid, and LysoPC(20:0)] were found to be significantly associated with Firmicutes disorder and may serve as potential markers of microbiota-gut-brain axis in the pathogenesis of MDD (Bai et al., 2021). The rs6265 variant in the BDNF gene was significantly associated with depression severity in untreated MDD patients (mean difference in HAMD-17 score 2.33, *p* = 0.014), suggesting that this genotype may serve as a genetic marker for disease severity, but no direct association with serum BDNF levels was observed (Losenkov et al., 2020). MicroRNAs are involved in the pathogenesis of MDD by regulating neuroinflammation, BDNF and HPA axis. Their abnormal expression profiles have diagnostic and therapeutic targeting value, providing epigenetic intervention strategies for precise diagnosis and treatment of MDD (Ortega et al., 2021). Prodan-Bărbulescu et al. (2024) found that 11 miRNAs such as hsa-miR-874- 3p, hsa-let-7d-5p and hsa-miR-93- 3p were significantly upregulated in plasma of MDD patients, which also proved their potential as a biomarker combination for MDD diagnosis.

Although existing studies have identified multiple potential biomarkers for MDD, clinical applications are still faced with insufficient diagnostic specificity of single markers and lack of standardized detection methods. Future studies need to focus on key issues such as marker specificity validation, detection method standardization, and multimodal combination model development to drive a paradigm shift in MDD diagnosis from symptom assessment to objective biological testing.

### 5.4 Biomarkers and diagnostic tools

Recent advances have begun to translate microglial insights into clinically useful markers for MDD. Elevated peripheral levels of IL-6, TNF-α and C-reactive protein closely mirror brain microglial activation and predict symptom severity, while measurements of soluble TREM2 in plasma offer a more microglia-specific readout. TSPO PET imaging reveals heightened ligand binding in the anterior cingulate cortex and hippocampus of depressed patients, providing *in vivo* mapping of neuroinflammation. At the same time, dysbiosis characterized by decreased Faecalibacterium and Bifidobacterium species aligns with both peripheral cytokine elevations and behavioral measures in preclinical models. More recently, microglia-derived exosomal miRNAs have emerged as blood-based proxies of central immune states. Integrative diagnostic platforms that merge cytokine panels, neuroimaging metrics, microbiome profiles and exosomal signatures are now under development, with the goal of stratifying patients into inflammation-driven versus non-inflammatory subtypes and selecting those most likely to benefit from microglia-modulating interventions. In TSPO PET work, Joo et al. included only 16 MDD patients without rs6971 genotyping, introducing affinity bias (Joo et al., 2021). Richards et al. (2018) controlled for genotype but pooled first-episode and recurrent cases, confounding disease stage effects. These methodological issues highlight the necessity for larger, genotype-balanced samples with rigorous clinical staging in future PET studies. Soluble TREM2 assays vary markedly across ELISA platforms, undermining comparability. TSPO PET is confounded by the rs6971 polymorphism, which alters ligand binding affinity and necessitates genotype-specific quantification. Emerging exosomal miRNA signatures lack replication in independent cohorts and suffer from low RNA yields. Harmonized assay standards, larger multi-center validation studies and combined PET-CSF biomarkers will be required to establish robust, clinically actionable diagnostics. Preclinical PET imaging in rodent models of depression shows robust upregulation of TSPO binding in hippocampus and cortex following chronic stress. However, human TSPO PET studies in MDD report mixed findings: some report modest increases in anterior cingulate TSPO signal, whereas others find no difference or even decreases when controlling for genotype. These inconsistencies likely reflect lower magnitude of microglial activation in humans, rs6971 polymorphism effects on ligand affinity, and variability in patient selection. Harmonized imaging protocols and larger samples stratified by genotype will be essential to resolve these discordant observations.

### 5.5 Microglia-mediated white-matter repair

Emerging evidence positions microglia as key regulators of myelin integrity in MDD. In addition to their well-established roles in synaptic pruning, activated microglia release trophic factors—most notably IGF-1, IGF-2 and BDNF—that drive oligodendrocyte progenitor cell proliferation and differentiation. Animal models of chronic stress demonstrate that enhancing microglial IGF signaling accelerates remyelination in the prefrontal cortex, restores conduction velocity and ameliorates depressive-like behaviors. Furthermore, microglial receptors such as TREM2 and CX3CR1 orchestrate phagocytic clearance of myelin debris, creating a permissive environment for oligodendrocyte recruitment and sheath rebuilding. Mapping the temporal dynamics of these pathways during stress and recovery will reveal optimal windows for intervention. Potential strategies include small-molecule TREM2 agonists, IGF-receptor modulators or microbiome-based SCFA supplementation to boost endogenous remyelination. Incorporating white-matter endpoints into future clinical trials will be essential to validate microglia-targeted remyelination as a novel therapeutic avenue for MDD. Trophic remyelination data to date derive mostly from young adult rodents, whereas MDD patients are typically middle-aged or older, raising questions about translatability. Human TREM2 agonists and IGF-receptor modulators are not yet clinically available, and diffusion tensor imaging lacks the specificity to distinguish myelin repair from axonal changes. Future efforts should include aged-animal cohorts, early-phase trials of remyelination-promoting agents, and multimodal MRI (e.g., DTI plus magnetization transfer ratio) to quantify microglia-mediated changes in white-matter integrity. As shown in Table 2, these comparisons highlight how microglia-mediated remyelination mechanisms extend beyond canonical inflammatory pathways and point to novel diagnostic and therapeutic avenues.

**TABLE 2:** Canonical vs. microglia-mediated white-matter repair mechanisms in MDD.

Aspect	Canonical view	Proposed white-matter repair framework	Key studies and testable directions	Clinical/diagnostic implications
Microglial role	Synaptic pruning; pro-inflammatory cytokine release	Secretion of trophic factors (IGF-1, BDNF); debris clearance via TREM2- and CX3CR1-dependent phagocytosis	IGF-1 release enhances OPC differentiation; TREM2 agonists promote myelin debris clearance	DTI+MTR imaging to track remyelination; CSF IGF-GFR imaging to
Oligodendrocyte interaction	Secondary damage from inflammation	Direct support of oligodendrocyte progenitor cells (OPCs) via microglial-derived exosomes containing miR-219	microglial exosomes rich in miR-219 drive OPC maturation	Exosomal miR-219 levels as predictive marker for therapy response
Signaling pathways	NF-κB, NLRP3 inflammasome activation	Activation of PI3K/Akt via IGF receptor; suppression of pro-apoptotic signals	PI3K/Akt blockade abrogates microglia-mediated remyelination *in vitro*	PET ligands for Akt phosphorylation in white matter
Temporal dynamics	Acute activation → chronic inflammation	Biphasic response: initial pro-inflammatory phase followed by reparative phase driven by M2-like microglia	time-course analysis of M2 markers (Arg-1, IL-10) in demyelination model	Longitudinal MRI to correlate M2 marker peaks with myelin recovery
Therapeutic strategies	Anti-inflammatories; monoamine modulation	TREM2 agonists; IGF receptor modulators; SCFA-enhancing probiotics	TREM2 agonist X improves myelin thickness post-stress	Design clinical trials combining TSPO PET and DTI endpoints

## 6 Conclusion and future outlook

This article focuses on the function and characteristics of microglia and their role in the pathogenesis of MDD. Microglia, as the main immune cells of the brain, regulate the occurrence and development of MDD through their anti-inflammatory and pro-inflammatory characteristics. This is confirmed in the function and characteristics of microglia in MDD. Antidepressants act by modulating microglial activity, as previously discussed, both by adaptively promoting microglial activation in response to injury and disease-related signals in the early stages of MDD, and by inhibiting the maintenance of depression-like symptoms or pain states due to changes in genetic coding; It also includes inhibiting the pro-inflammatory response of M1 microglia, promoting the anti-inflammatory and repair effects of M2 microglia, regulating neurotrophic factors and neuronal plasticity, transforming the activated state of microglia into a resting state, reducing the inflammatory response caused by excessive activation, and exerting neuroprotective and antidepressant effects. These findings suggest that microglia may be a potential therapeutic target that influences the development of MDD through different phenotypes and metabolic states. Modulation of microglia function and properties may help reshape the CNS microenvironment, thereby improving therapeutic outcomes for patients with MDD.

However, we also found some limitations of microglia in MDD. First, most of the current studies are limited to the direct correlation between microglia and MDD, and play an antidepressant role by regulating the upstream substance to regulate the function of microglia. However, it is not known whether the upstream substance only regulates microglia, whether there is an upstream substance to regulate other aspects at the same time, and whether microglia plays a synergistic role with other glia. The specific mechanism is also unknown. Further reviews are needed to more fully understand the pathophysiological changes of microglia in MDD. Second, the mechanism of microglia in different sex, age and environmental conditions remains to be further studied. Although the relationship between microglia and sex and environmental factors in MDD has been pointed out, there are still some key mechanisms and causal relationships that need to be further confirmed. For example, how sex differences affect the regulatory mechanisms of brain sex differentiation, transcriptome and epigenetic changes in microglia during aging, and microglia responses to environmental factors and affect the specific mechanisms of MDD development will require more basic experimental results and clinical trial data to confirm. Finally, how to precisely regulate the polarization of microglia functional characteristics and translate these findings into clinical studies will be key directions for future research.

## 7 New assumptions

**FIGURE 7:**
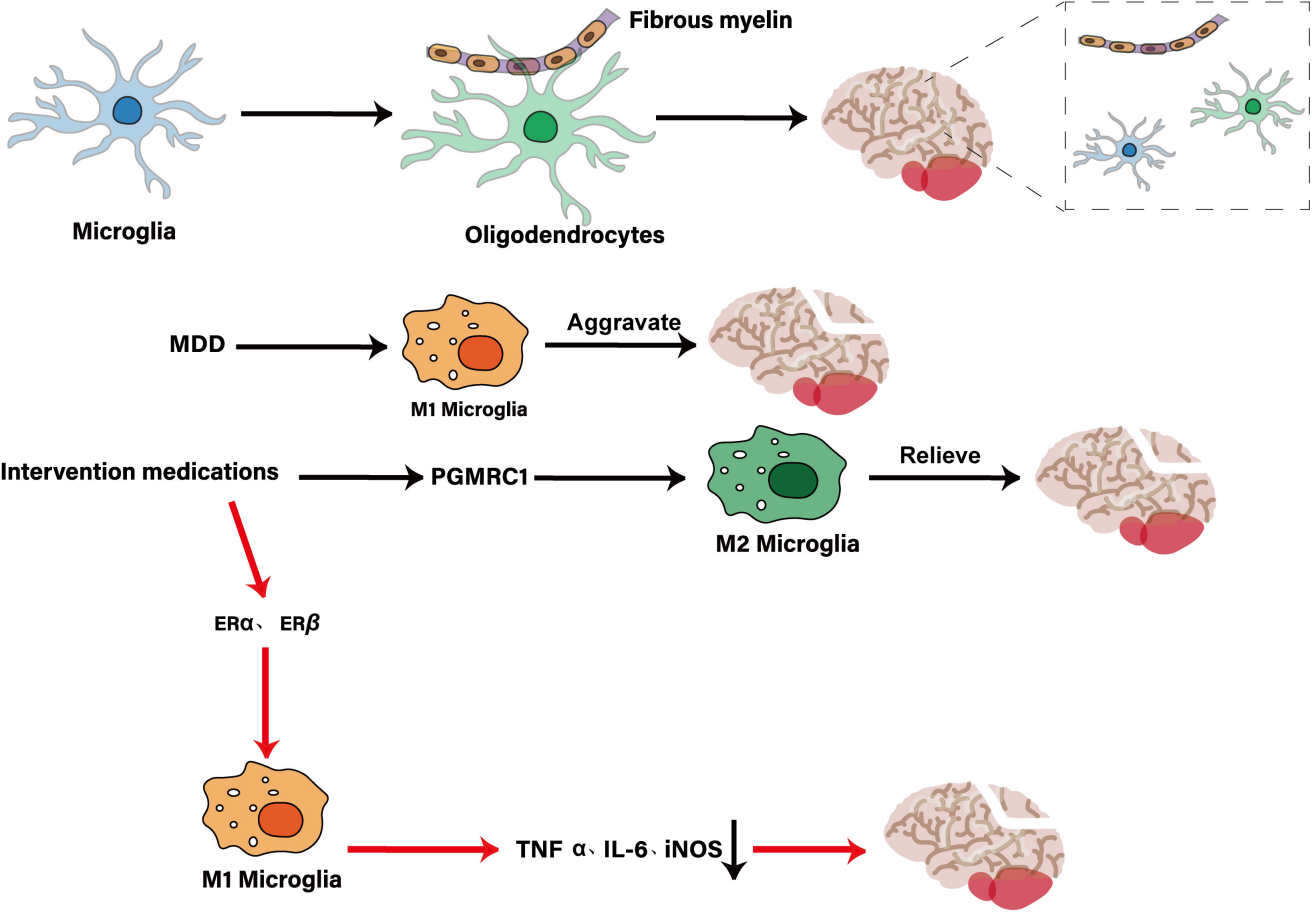
Mechanism diagram of the microglia-oligodendrocyte-white matter repair hypothesis. The figure shows an increase in pro-inflammatory microglia in the context of MDD, exacerbating white matter damage. Intervention drugs can reduce white matter damage and promote the recovery of cognitive function in chronic recovery by regulating microglia function, which depends on the regulation of microglia function by intervention drugs. On the one hand, it regulates the neurosteroid receptor PGMRC1 pathway in microglia, promotes the transformation of microglia into repair, increases the expression of tissue repair factors (such as CD206, TGM2, VEGFA, TIMP1, FGF1, PTN), and promotes repair. On the other hand, it regulates ERα and ERβ pathways, inhibits the transformation of microglia into pro-inflammatory types, reduces the expression of pro-inflammatory factors (such as TNFα, IL-6, iNOS, etc.), and inhibits damage.

Recent studies have found that the “microglial-oligodendrocyte-white matter repair” pathway may be a new way to improve inflammatory damage in MDD. Diffusion tensor imaging (DTI) technology has found that patients with MDD have significant microstructural changes in white matter, with reduced anisotropy scores of white matter fiber tracts (such as corpus callosum and corona radiata) and impaired fiber myelination (Giera et al., 2018). White matter is composed mainly of myelinated fibers and glial cells, which are responsible for connecting neurons in various regions of the brain and play a vital role in neural signal transmission and interbrain communication. Microglial cells can promote myelination and repair by regulating the proliferation of oligodendrocyte precursor cells (Song et al., 2022). Under pathological conditions, microglia activation changes from pro-inflammatory microglia to tissue repair microglia, which is essential for white matter repair and functional recovery. Studies have shown (Lei et al., 2014) that by regulating the polarization of microglia, it can promote oligodendrocyte generation and myelination, thus improving neural function. The polarization of microglia has a significant impact on the repair process after nerve injury. M1 microglia release pro-inflammatory factors, which may aggravate nerve injury, while M2 microglia release anti-inflammatory factors, which promote tissue repair and functional recovery. Modulating microglia polarization toward M2 by drugs or other treatments may be a novel strategy for treating MDD. Neurosteroid receptors on microglia also play an important role in regulating microglia function. For example, estrogen ERα and ERβ pathway reduce the proinflammatory response of microglia, while progesterone receptor PGMRC1 pathway increases the repair function of microglia. Mouse experiments have confirmed that progesterone secreted by astrocytes and oligodendrocytes binds to PGMRC1 receptor on microglia and increases the expression of anti-inflammatory factors (Yilmaz et al., 2019). *In vitro* experiments have shown that increasing PGMRC1 expression can upregulate the level of anti-inflammatory factors in microglia under hypoxic conditions, thus promoting white matter repair (Gargiulo-Monachelli et al., 2019). Based on this, the authors hypothesized that, as shown in Figure 7, in the context of MDD, proinflammatory glia increased, exacerbating white matter damage. Interventional drugs can reduce white matter damage and promote cognitive recovery in chronic recovery period by regulating microglia function, which depends on the regulation of microglia function by interventional drugs. On the one hand, it regulates the neurosteroid receptor PGMRC1 pathway in microglia, promotes the transformation of microglia into repair type, and increases tissue repair factors (such as CD206, TGM2, VEGFA, TIMP1, FGF1, PTN) expression, promote repair; on the other hand, regulate ERα, ERβ pathway, inhibit microglia to pro-inflammatory transformation, reduce pro-inflammatory factors In conclusion, microglia play a complex and diverse role in maintaining CNS homeostasis and participating in MDD, which deserves further exploration and study.
